# Familywise error control in multi‐armed response‐adaptive trials

**DOI:** 10.1111/biom.13042

**Published:** 2019-04-03

**Authors:** D. S. Robertson, J. M. S. Wason

**Affiliations:** ^1^ MRC Biostatistics Unit University of Cambridge IPH Forvie Site, Robinson Way Cambridge CB2 0SR UK; ^2^ Institute of Health and Society Newcastle University Newcastle upon Tyne NE2 4AX UK

**Keywords:** Bayesian methods, closed testing, multiple comparisons, response‐adaptive randomization, type I error

## Abstract

Response‐adaptive designs allow the randomization probabilities to change during the course of a trial based on cumulated response data so that a greater proportion of patients can be allocated to the better performing treatments. A major concern over the use of response‐adaptive designs in practice, particularly from a regulatory viewpoint, is controlling the type I error rate. In particular, we show that the naïve *z*‐test can have an inflated type I error rate even after applying a Bonferroni correction. Simulation studies have often been used to demonstrate error control but do not provide a guarantee. In this article, we present adaptive testing procedures for normally distributed outcomes that ensure strong familywise error control by iteratively applying the conditional invariance principle. Our approach can be used for fully sequential and block randomized trials and for a large class of adaptive randomization rules found in the literature. We show there is a high price to pay in terms of power to guarantee familywise error control for randomization schemes with extreme allocation probabilities. However, for proposed Bayesian adaptive randomization schemes in the literature, our adaptive tests maintain or increase the power of the trial compared to the *z*‐test. We illustrate our method using a three‐armed trial in primary hypercholesterolemia.

## 1 INTRODUCTION

Clinical trials typically randomize patients using a fixed randomization scheme, where the probabilities of assigning patients to the experimental treatments and control are pre‐specified and constant. A common method is to simply use equal randomization to the different arms of the trial. However, such randomization schemes can mean that a substantial proportion of the trial participants will continue to be allocated to treatments that are not the best available, even if interim data indicates that one treatment is likely to be superior. Response‐adaptive trials address this concern by adaptively changing the randomization probabilities, so that a greater proportion of patients are allocated to the treatment arm which has a better performance based on the cumulated response data.

Many classes of response‐adaptive randomization (RAR) schemes have been proposed for binary outcomes, and there is also a growing interest in RAR for continuous responses. In Web Appendix A, we present an overview of multi‐arm RAR schemes described in the literature. Our focus in this article is on normally distributed outcomes, which are encountered in a number of clinical trials. Indeed, 23 out of the 59 trials identified in a review of multi‐arm trials by Wason et al. (2014) had a continuous outcome.

Despite the extensive literature on RAR, relatively few clinical trials have actually used such schemes in practice. A current example is the ongoing I‐SPY 2 trial (Park et al., 2016), which screens drugs in neoadjuvant breast cancer and uses RAR as part of its design. One of the key concerns over using RAR, particularly from a regulatory perspective, is ensuring that the type I error rate is controlled. In a multi‐arm trial, multiple hypotheses are tested simultaneously by design, which leads to a multiple testing problem. To account for this, testing procedures are used that guarantee strong control of the familywise error rate (FWER), which ensures the maximum probability of making at least one type I error is controlled. For confirmatory trials in particular, demonstrating strong control of the FWER is often required by regulators (European Medicines Agency, 2002; Food and Drug Administration, 2018).

For response‐adaptive trials, a rigorous proof of FWER control for a particular design is difficult given the complexities of the treatment allocation process. Hence error control has typically either been demonstrated through simulation studies, or by exploiting the asymptotic structure of the adaptive randomization procedure (Hu and Rosenberger, 2006; Zhu and Hu, 2010). However, neither method provides a guarantee of FWER control, particularly with small sample sizes. Another possibility is the use of (re)'randomization tests to preserve type I error (Simon and Simon, 2011), which we return to in Section 6. Gutjahr et al. (2011) showed how to achieve strong control of the FWER for normally distributed outcomes in a two‐stage design incorporating RAR in the first stage. However, our focus is on general response‐adaptive trials, without the necessity of restricting to two stages or having a final stage of equal randomization.

In this article, we show how to guarantee strong control of the FWER for both fully sequential and block randomized response‐adaptive trials, for a large class of adaptive randomization rules. Our proposed procedure works by reweighting the usual *z*‐statistic through an iterative application of the conditional invariance principle. The resulting test statistic can then be used to test the null hypothesis that a treatment is superior to the control.

The rest of the article is organised as follows. In Section 2, we describe the proposed method for fully sequential response‐adaptive trials with a fixed allocation to the control. This method is then modified for block randomized response‐adaptive trials in Section 3, for both a fixed or adaptive control allocation. Simulation studies for the proposed methods are presented in Section 4, and Section 5 gives a case study based on a trial in primary hypercholesterolemia. We conclude with a discussion in Section 6. All proof details can be found online in the Supporting Information section.

## 2 FULLY SEQUENTIAL RESPONSE‐ADAPTIVE TRIALS

### 2.1 Trial setting

Suppose a trial is conducted to test t>1 experimental treatments against a common control, using the following design. A total of *n* patients are allocated to the experimental treatments, and $n_{0}$ patients are allocated to the control, where $n_{0}$ and *n* are fixed in advance. Patients are allocated to the different experimental treatments using RAR, where we assume that the randomization rule does not depend on the control information. We also assume the allocation to the control is fixed; that is, the probability of assigning a patient to the control is pre‐specified and constant. Maintaining allocation to the control is recommended by the Food and Drug Administration (2018), since it best maintains the power of the trial, and helps address the concern about changing patient characteristics over the course of the trial.

The RAR for the experimental treatments starts with a burn‐in period *B*, which uses fixed randomization to allocate $r_{i}>0$ patients to the *i*th treatment (i=1,…,t), with the $r_{i}$ again fixed in advance. Hence a total of $r=\sum_{i=1}^{t}r_{i}$ patients are allocated to the experimental treatments during the burn‐in period. Let $a_{k}$ denote the treatment allocation for the *k*th experimental patient (k=1,…,n), where $a_{k}=i$ if the *k*th patient is allocated to the *i*th treatment. The allocation $a_{k}$ can depend on the data (i.e., the allocations and outcomes) observed up to patient k−1, as well as any external information available at that time. Also let $X_{k}$ denote the efficacy outcome for the *k*th patient, while $X_{0j}$ denotes the efficacy outcomes for the *j*th patient on the control ($j=1,\ldots ,n_{0}$) where

$$X_{0j}∼N(\mu ,\sigma^{2}),\;\;X_{k}\;{|}_{a_{k}=i}\;∼N(\mu +\delta_{i},\sigma^{2})$$

The variance $\sigma^{2}$ is assumed known and, without loss of generality, we set $\sigma^{2}=1$. Here $\delta_{i}$ represents the incremental benefit of treatment *i* compared to the control, and is the parameter of interest. Finally, let $n_{i}$ denote the total number of allocations to the *i*th experimental treatment (including the burn‐in period) and $n_{I}=\sum_{i\in I}n_{i}$.

### 2.2 Hypothesis testing

The elementary null hypotheses are $H_{i}:\delta_{i}=0$ against the one‐sided alternatives ${\bar{H}}_{i}:\delta_{i}>0$. We discuss the case when $H_{i}:\delta_{i}\leq 0$ at the end of Section 2.5. One general method to control for multiple testing is to use the closure principle (Marcus et al., 1976) and consider all intersection hypotheses $H_{I}$, where I⊆{1,…,t}. To strongly control the FWER, we reject an elementary null hypothesis $H_{i}$ if we also reject every $H_{I}$ with i∈I using a local level’α test. Hence we need to define a valid level’α test for all the intersection hypotheses $H_{I}$.

The naïve *z*‐test for $H_{I}$, which does not take into account the RAR used in the trial, rejects $H_{I}$ if the test statistic

$$T_{I}=\sum_{k=1}^{n}\left(1_{\left\{a_{k}\in I\right\}}\frac{X_{k}}{n_{I}}\right)-\sum_{j=1}^{n_{0}}\frac{X_{0j}}{n_{0}}$$

is greater than $z_{\alpha }{\left(1/n_{I}+1/n_{0}\right)}^{1/2}$, where $z_{\alpha }$ is the (1−α) standard normal quantile.

As an alternative to using the closure principle with the test statistic above, one could simply use the Bonferroni correction, or a step‐up/step‐down procedure such as the Holm procedure. These would only involve calculating test statistics for the *t* elementary null hypotheses, i.e., calculating $T_{I}$ for I={i} (i=1,…,t). Hence we present the methodology assuming the closure principle will be used, with the Bonferroni and Holm procedures considered as special cases. We return to this issue in Section 4.

### 2.3 Inflation of the familywise error rate

Since the *z*‐test ignores the adaptive randomization used, it is possible to inflate the FWER. As an example, consider the following adaptive randomization scheme for t=2 treatments:

$$a_{k+1}=\left\{\begin{array}{cc} 2 & \mathrm{if}\;\;\sum_{j=1}^{k}(1_{\left\{a_{j}=1\right\}}\frac{X_{j}}{n_{1k}})>0.5\\ 1 & \mathrm{otherwise} \end{array}\right.$$

where $n_{1k}=\sum_{j=1}^{k}1_{\left\{a_{j}=1\right\}}$. This can be viewed as implementing early stopping for efficacy for treatment 1, which is not taken into account using the naïve *z*‐test.

We ran a simulation study to calculate the type I error rate using the above randomization scheme. We set α=0.05, $n_{0}=n=60$, $r_{1}=r_{2}=5$ and the true treatment means $\mu =0,\delta_{1}=0$, $\delta_{2}=1$. The type I error rate as averaged over $10^{5}$ simulations is 10.4%, more than double the nominal 5% level. We subsequently refer to allocation rules of this type as ‘type I error inflator’ rules (which clearly would never be used in practice).

### 2.4 Auxiliary design

Working with the actual design of the trial is difficult because RAR affects the distribution of the usual *z*‐test statistics. Hence for each $H_{I}$ we introduce a simpler design, called the auxiliary design, for which we do know the distribution. The actual trial design can then be viewed as a series of data‐dependent modifications of the auxiliary design, where we account for the modifications using the conditional invariance principle. The auxiliary designs are purely hypothetical, and are only used to construct the modified tests for the actual design. As well, the allocations in the auxiliary designs are fixed before the start of the actual trial.

The auxiliary design for hypothesis $H_{I}$ is as follows. As in the actual design, a total of *n* patients are allocated to the experimental treatments, and $n_{0}$ patients are allocated to control. The allocations and responses to the control treatment are the same as the actual design. For the patients allocated to the experimental treatments, the auxiliary design starts with a burn‐in period *B* with *r* patients that is identical to the actual design. The subsequent n−r−1 allocations are given by a fixed sequence $\left(b_{r+1},\ldots ,b_{n-1}\right)$, which can be chosen arbitrarily. The final allocation $b_{n}$ must be to one of the treatments in *I*.

These allocations can be considered as an approximation, made *before the trial begins*, to what we could reasonably expect the actual allocations to be. Assuming clinical equipoise, there is no way of knowing *a priori* that the probability of assignment to a treatment will be higher or lower than that for any other treatment (given some adaptive randomization scheme). Hence randomizing equal numbers of patients to each treatment for the auxiliary design is the natural choice for trials where there is genuine uncertainty over the effectiveness of the different treatments. We return to these issues in Section 6.

For the auxiliary design, let $Y_{k}$ denote the efficacy outcome for the *k*th patient under the auxiliary design (k=1,…,n), where $Y_{k}=X_{k}$ for k=1,…,r by design. Also let $n_{i}^{\prime }=\sum_{j=1}^{n}1_{\left\{b_{j}=i\right\}}$ denote the total number of allocations to the *i*th experimental treatment, and $m_{i,k}=\sum_{j=k}^{n}1_{\left\{b_{j}=i\right\}}$ denote the total number of allocations to the *i*th treatment for patients (k,k+1,…,n). We then define $n_{I}^{\prime }=\sum_{i\in I}n_{i}^{\prime }$ and $m_{I,k}=\sum_{i\in I}m_{i,k}$. Under the auxiliary design, $n_{i}^{\prime }$ is fixed for all *i*, and hence under $H_{I}$, the usual *z*‐statistic

$$T_{I}^{\prime }=\sum_{k=1}^{n}\left(1_{\left\{b_{k}\in I\right\}}\frac{Y_{k}}{n_{I}^{\prime }}\right)-\sum_{j=1}^{n_{0}}\frac{X_{0j}}{n_{0}}$$

is normally distributed with mean zero and variance $\left(1/n_{I}^{\prime }+1/n_{0}\right)$. Hence we reject $H_{I}$ if $T_{I}^{\prime }$ is greater than $z_{\alpha }{\left(1/n_{I}^{\prime }+1/n_{0}\right)}^{1/2}$.

### 2.5 Adaptive test statistic

Adaptive designs, such as the trial being considered, follow a common conditional invariance principle in order to control the type I error rate (Brannath et al., 2007). For our response‐adaptive trial in question, we apply the conditional invariance principle sequentially, where each step considers the next patient recruited into the trial. Below we give the test statistic for testing hypothesis $H_{I}$ under the actual design, given that the allocation is fully sequential.

Theorem 1 Under $H_{I}$, the following test statistic is normally distributed with mean 0 and variance $\left(1/n_{I}^{\prime }+1/n_{0}\right)$

$${\tilde{T}}_{I}=\sum_{k=1}^{n}\left(1_{\left\{a_{k}\in I\right\}}\frac{X_{k}}{w_{k}^{\left(I\right)}}\right)-\sum_{j=1}^{n_{0}}\frac{X_{0j}}{w_{n,j}^{\left(0\right)}}$$

The weights $w_{k}^{\left(I\right)}$ and $w_{n,j}^{\left(0\right)}$ for the efficacy outcomes are calculated recursively based on the number of allocations to the experimental treatments and the control, with full details and the proof of Theorem 1 provided in Web Appendix B.

Using Theorem 1, we reject $H_{I}$ if ${\tilde{T}}_{I}$ is greater than $z_{\alpha }{\left(1/n_{I}^{\prime }+1/n_{0}\right)}^{1/2}$. In practice, to keep the weights as close to the natural weight $n_{0}$ for as many of the control observations as possible, we recommend setting $m_{0,1}=n_{0}-1$ and $m_{0,2}=1$, as used for the simulation studies in Section 4.1. As a simple illustration of how the weights change over the course of a trial, consider testing t=2 experimental treatments. We set α=0.05, $n_{0}=10$, n=11 and $r_{1}=r_{2}=1$. Suppose we have no a priori reason to favour one treatment over the other, and so we simply choose the auxiliary design to be an equal randomization of the two treatments:

$$b=\begin{array}{cccccccccccccccccccccc} 1 & 2 & 2 & 1 & 2 & 2 & 1 & 1 & 2 & 1 & * & & & & & & & & & & & \end{array}$$

Here the vertical line indicates where the burn‐in period ends, and the * represents the allocation for $b_{n}$, which by design must satisfy $b_{n}\in I$. We set $m_{0,1}=9$ and $m_{0,2}=1$, so that $w_{1}^{\left(0\right)}=\cdots =w_{9}^{\left(0\right)}$. Table 1 below shows how the weights change over the course of a trial for an actual allocation *a* that is similar to the auxiliary design *b*. Further examples for a variety of actual allocations *a* can be found in Web Appendix C.

**Table 1:** An actual allocation *a* that is similar to the auxiliary design *b*. The weights that would be used in the naïve *z*‐test are $n_{0}=10$, $n_{1}=4$ and $n_{2}=7$

*a* =	1	2	2	2	1	2	2	1	2	1	2	
*b* =	1	2	2	1	2	2	1	1	2	1	*	
$w^{\left(1\right)}$ =	6	6	6	5.16	6	6	4.94	4.94	4.94	4.94	–	$w_{1}^{\left(0\right)}=9.74,w_{10}^{\left(0\right)}=-5.38$
$w^{\left(2\right)}$ =	6	6	6	7.01	5.74	5.74	7.63	7.63	7.63	7.63	7.63	$w_{1}^{\left(0\right)}=w_{10}^{\left(0\right)}=9.58$

In all of the scenarios that we have investigated, the weights $w_{k}^{\left(I\right)}$ for the experimental treatments have been positive (although we cannot rule out the possibility of having negative or imaginary weights). Hence in these cases, the test procedure also controls the FWER for the composite null hypotheses $H_{i}:\delta_{i}\leq 0$. To see this, suppose the elementary null hypotheses are $H_{i}^{*}:\delta_{i}=\delta_{i}^{*}<0$. Under $H_{I}^{*}$, we can rewrite the distribution of the responses $X_{k}^{*}$ as $X_{k}+\delta_{k}^{*}$, where $X_{k}∼N(0,1)$. Hence under $H_{I}^{*}$

$$\mathrm{pr}({\tilde{T}}_{I}^{*}>c)=\mathrm{pr}\left({\tilde{T}}_{I}>c-\sum_{k=1}^{n}1_{\left\{a_{k}\in I\right\}}\frac{\delta_{k}^{*}}{w_{k}^{\left(I\right)}}\right)<\mathrm{pr}({\tilde{T}}_{I}>c)$$

where ${\tilde{T}}_{I}^{*}$ and ${\tilde{T}}_{I}$ are the adaptive test statistics for $H_{I}^{*}$ and $H_{I}$ respectively.

## 3 BLOCK RANDOMIZED RESPONSE‐ADAPTIVE TRIALS

### 3.1 Trial setting

It may not be feasible or desirable to randomize patients one‐by‐one in a fully sequential manner. Instead one can use block randomization, where after the burn‐in period *B*, patients are adaptively randomized to the experimental treatments in blocks of size $\left(d_{1},\ldots ,d_{J}\right)$ over *J* stages, with $\sum_{j=1}^{J}d_{j}=n$. The randomization of the *j*th block depends on the data up to block (j−1), as well as any external information available at the time. The allocation to the control is again assumed to be fixed throughout the trial.

Due to the block structure of the trial, we can relax the assumption that the randomization rule used for the experimental treatments does not depend on the control information. This is achieved by splitting up the $n_{0}$ patients allocated to the control into blocks. More explicitly, suppose that during the burn‐in period, $r_{0}>0$ patients are allocated to the control, where $r_{0}$ is fixed in advance. Subsequently, in the *j*th block, $d_{0j}$ patients are allocated to the control, where $\sum_{j=1}^{J}(r_{0}+d_{0j})=n_{0}$. We assume that for the final block $d_{0J}>1$. The response‐adaptive randomization at block *l* may now depend on the control information available at the end of block (l−1); that is, the outcome data available from the first $\sum_{j=1}^{l-1}(r_{0}+d_{0j})$ patients allocated to the control.

To control the FWER, we can modify the approach in Section 2 to account for the block structure. As before, we have an auxiliary design for the patients on the experimental treatments, but now in step *l* of the process the actual design is a data‐dependent modification of all the allocations for the patients in block *l*. Hence the weights for the observations in each block will be the same, and are updated block‐by‐block. Full details of the auxiliary design and the resulting adaptive test statistic ${\tilde{T}}_{I}$ can be found in Web Appendix D.

### 3.2 Extension for adaptive control allocations

Thus far, we have assumed that the allocations to the control follow some fixed scheme. We now relax this assumption in the block‐randomized setting. The formula for the adaptive test statistic ${\tilde{T}}_{I}$ can be found in Web Appendix E. Note that it is possible the procedure will fail to give a valid test statistic in this setting, as shown in Web Appendix F.3.

## 4 SIMULATION STUDIES

As we have already seen in Section 2.3, using the closure principle with the usual *z*‐test does not strongly control the FWER. An alternative method is to use the Bonferroni correction on the elementary null hypotheses $H_{1},\ldots ,H_{t}$. We also consider the Holm procedure, which is a step‐down procedure that is uniformly more powerful than Bonferroni (Holm, 1979). An advantage of both these procedures is that only *t* test statistics are calculated, rather than $\left(2^{t}-1\right)$ test statistics when using the closure principle. This motivates also applying the Holm procedure to the *p*‐values derived from the adaptive test statistics ${\tilde{T}}_{i}$ for i=1,…,t. More precisely, we use the adjusted *p*‐values ${\tilde{p}}_{i}=1-\Phi ({\left(1/n_{i}^{\prime }+1/n_{0}^{\prime }\right)}^{-1/2}\;{\tilde{T}}_{i})$, instead of the usual *p*‐values $p_{i}=1-\Phi ({\left(1/n_{i}+1/n_{0}\right)}^{-1/2}\;T_{i})$ derived from the *z*‐test.

To distinguish between the methods, we call our proposed procedure that uses the closure principle the ‘adaptive closed test’. Similarly, applying the closure principle to the usual *z*‐test gives the ‘closed *z*‐test’. Applying the Holm procedure to our adjusted *p*‐values gives the ‘Holm adaptive test’, while applying the Holm procedure to the usual *p*‐values gives the ‘Holm *z*‐test’. In our simulation studies, we compare the different methods by looking at the FWER and the power of the different tests. To keep the comparisons simple, and as a similar measure to the FWER, we present results for the disjunctive power, which is the probability of rejecting at least one false null hypothesis. In order to see how the adaptive randomization procedures affect power, we provide comparisons with using equal randomization in Web Appendix F.5.

### 4.1 Fully sequential randomization

We first consider a fully sequential response‐adaptive trial, as presented in Section 2, with m=50 patients allocated to the experimental treatments after the burn‐in and $n_{0}=60/t$ patients allocated to the control. In the burn‐in period, five patients are allocated to each of the experimental treatments. We set α=0.05 and the true control mean μ=0 for simplicity. We compare the methods under two randomization schemes, the first being the Type I error inflator and the second being a Bayesian Adaptive Randomization (BAR) scheme, with full details given in Web Appendix F.1.

*Simulation results*: Table 2 gives the results for the type I error inflator randomization scheme, while Table 3 gives the results for BAR. The auxiliary designs in all scenarios were simply (m−1) random draws from a discrete uniform distribution on {1,…,t}.

**Table 2:** Familywise error rate and disjunctive power for the type I error inflator in the fully sequential setting. There were $10^{5}$ simulated trials for each set of parameter values

		Adaptive closed test		Adaptive test (Holm)		Closed *z*‐test		*z*‐test (Holm)		*z*‐test (Bonferroni)	
	Parameter values	Error	Power	Error	Power	Error	Power	Error	Power	Error	Power
1.	$\delta_{1}=\delta_{2}=0$	3.3	–	4.7	–	4.7	–	**7.0**	–	**7.0**	–
2.	$\delta_{1}=0$, $\delta_{2}=1$	4.8	21.7	3.7	27.5	**10.3**	26.5	**9.9**	63.6	5.0	63.5
3.	$\delta_{1}=\delta_{2}=0.5$	–	62.4	–	52.4	–	69.9	–	61.6	–	61.6
4.	$\delta_{1}=\delta_{2}=\delta_{3}=0$	2.8	–	3.8	–	4.1	–	**5.9**	–	**5.9**	
5.	$\delta_{1}=\delta_{2}=0$, $\delta_{3}=1$	3.2	13.1	4.2	24.2	**5.1**	17.2	**6.4**	54.2	4.5	54.1
6.	$\delta_{1}=0$, $\delta_{2}=\delta_{3}=1$	4.6	22.2	3.2	28.0	**9.7**	27.0	**9.0**	75.4	3.2	75.4
7.	$\delta_{1}=0$, $\delta_{2}=0.5$, $\delta_{3}=1$	4.0	19.1	2.6	24.5	**9.1**	23.9	**7.4**	58.5	3.2	58.4
8.	$\delta_{1}=\delta_{2}=\delta_{3}=0.5$	–	51.3	–	41.7	–	57.8	–	49.7	–	49.7

**Table 3:** Familywise error rate and disjunctive power for BAR in the fully sequential setting. There were $10^{5}$ simulated trials for each set of parameter values

		Adaptive closed test		Adaptive test (Holm)		Closed *z*‐test		*z*‐test (Holm)		*z*‐test (Bonferroni)	
	Parameter values	Error	Power	Error	Power	Error	Power	Error	Power	Error	Power
1.	$\delta_{1}=\delta_{2}=0$	4.7	–	4.5	–	4.8	–	4.1	–	4.1	–
2.	$\delta_{1}=0$, $\delta_{2}=0.5$	4.6	46.4	4.4	52.4	3.9	46.7	3.6	53.6	1.9	53.5
3.	$\delta_{1}=\delta_{2}=0.5$	–	70.8	–	66.4	–	71.2	–	65.9	–	65.9
4.	$\delta_{1}=\delta_{2}=\delta_{3}=0$	3.8	–	4.1	–	4.0	–	3.8	–	3.8	
5.	$\delta_{1}=\delta_{2}=0$, $\delta_{3}=1$	4.4	59.9	4.2	88.7	4.3	60.1	3.8	90.6	2.6	90.6
6.	$\delta_{1}=0$, $\delta_{2}=\delta_{3}=1$	4.8	89.8	4.7	95.1	4.0	90.1	3.9	96.0	1.3	96.0
7.	$\delta_{1}=0$, $\delta_{2}=0.5$, $\delta_{3}=1$	4.3	74.8	3.9	88.2	3.9	75.7	3.4	90.0	1.4	90.0
8.	$\delta_{1}=\delta_{2}=\delta_{3}=0.5$	–	56.5	–	51.8	–	57.9	–	52.7	–	52.7

Looking first at the results for the type I error inflator in Table 2, the closed *z*‐test does not control the FWER in the scenarios where at least one null hypothesis is false, with an error rate as high as 10.3% in scenario 2. Applying the Holm procedure to the *z*‐test does not control the FWER, and actually increases the error rate in some scenarios (1 and 4). Applying Bonferroni to the *z*‐test also does not control the FWER, as can be seen in scenarios where all null hypotheses are true. In contrast, both the adaptive closed test and the Holm adaptive test strongly control the FWER, although they tend to be rather conservative.

The fact that the Bonferroni correction, the use of the closure principle and the Holm procedure all do not control the FWER may appear surprising at first. The inflation occurs because when using RAR, the usual *z*‐test will no longer be normally distributed under the null. Therefore the *p*‐values derived from the *z*‐statistics are not guaranteed to be stochastically larger than or equal to the uniform distribution under the null, which is required for these methods to work.

As for the power of the different methods, when at least one of the null hypotheses is true (as in scenarios 2, 5, 6 and 7), the Holm *z*‐test has substantially higher power than the closed *z*‐test. Indeed, the power more than doubles in all four scenarios, and even more than triples in scenario 5. This dramatic increase in power demonstrates that in these scenarios, the closed *z*‐test is not very sensitive. This is because the test statistic for $H_{I}$ will be ‘diluted’ by the contribution from responses belonging to the null hypotheses ${\left(H_{i}\right)}_{i\in I}$ that are true. It is only when all of the null hypotheses are false, as in scenarios 3 and 8, that the power of the closed *z*‐test is reasonable, with a slightly higher power than the Holm *z*‐test.

As for the adaptive tests, the adaptive closed test has a slightly lower power than the closed *z*‐test for all scenarios, with an absolute decrease of between 4.1% in scenario 5 and 7.5% in scenario 3. However, the Holm adaptive test has a substantially lower power than the Holm *z*‐test, with the latter having more than double the power. This demonstrates the high cost in terms of power that controlling the FWER can incur for this randomization scheme. We return to this issue in Section 4.3.

Turning to the BAR scheme in Table 3, this time all of the methods strongly control the FWER. All methods are slightly conservative, with the adaptive closed test being generally the closest to the nominal 5% level. The Bonferroni‐corrected *z*‐test is noticeably more conservative than all the other methods, particularly when there are three treatments. In terms of disjunctive power, if at least one of the null hypotheses are true, we again see that the closed tests suffer from reduced power compared to the Holm versions. However, with BAR the loss of power is less dramatic, with a maximum of a 33% relative decrease in power in scenario 5, but with much smaller decreases in scenarios 2 and 7 for example. This time, the adaptive closed test has almost the same power as the closed *z*‐test, losing a maximum of only 1.4% in scenario 8. In addition, the Holm adaptive test and Holm *z*‐test now have comparable power, with a maximum loss of only 1.9% in scenarios 5 and 7. This indicates that for BAR schemes, the adaptive tests do not lose out very much in terms of power.

### 4.2 Block randomization with a fixed control allocation

We now consider block randomized trials with a fixed control allocation, as presented in Section 3.1. We use the setup of a trial with J=3 blocks, with sizes (40, 40, 40) for the experimental treatments and (20, 20, 20) for the control. In the burn‐in period, five patients are allocated to each of the treatments including the control. We set the true control mean μ=0, and α=0.05. We compare the methods under a type I error inflator scheme and a BAR scheme, with full details given in Web Appendix F.2.

*Simulation results*: Table 4 gives the results for the type I error inflator randomization scheme, while Table 5 gives the results for BAR. The auxiliary designs in all scenarios were simply random draws from a discrete uniform distribution on {1,…,t}.

**Table 4:** Familywise error rate and disjunctive power for the type I error inflator, for block randomization with a fixed control allocation. There were $10^{5}$ simulated trials for each set of parameter values

		Adaptive closed test		Adaptive test (Holm)		Closed *z*‐test		*z*‐test (Holm)		*z*‐test (Bonferroni)	
	Parameter values	Error	Power	Error	Power	Error	Power	Error	Power	Error	Power
1.	$\delta_{1}=\delta_{2}=0$	3.8	–	4.8	–	4.6	–	**6.5**	–	**6.5**	–
2.	$\delta_{1}=0$, $\delta_{2}=1$	4.8	22.0	3.6	26.9	**8.3**	25.6	**7.8**	61.1	4.3	61.0
3.	$\delta_{1}=\delta_{2}=0.5$	–	92.7	–	87.9	–	94.6	–	91.7	–	91.7
4.	$\delta_{1}=\delta_{2}=\delta_{3}=0$	3.2	–	4.1	–	4.1	–	**6.1**	–	**6.1**	
5.	$\delta_{1}=\delta_{2}=0$, $\delta_{3}=1$	3.7	14.2	4.4	23.4	4.7	18.1	**6.2**	61.2	4.5	61.1
6.	$\delta_{1}=0$, $\delta_{2}=\delta_{3}=1$	4.9	20.1	3.2	26.1	**8.1**	23.0	**7.3**	78.5	3.2	78.4
7.	$\delta_{1}=0$, $\delta_{2}=0.5$, $\delta_{3}=1$	4.7	17.7	3.0	23.8	**8.0**	21.1	**6.7**	66.2	2.8	66.2
8.	$\delta_{1}=\delta_{2}=\delta_{3}=0.5$	–	91.3	–	83.4	–	94.0	–	89.7	–	89.7

**Table 5:** Familywise error rate and disjunctive power for BAR, for block randomization with a fixed control allocation. There were $10^{5}$ simulated trials for each set of parameter values

		Adaptive closed test		Adaptive test (Holm)		Closed *z*‐test		*z*‐test (Holm)		*z*‐test (Bonferroni)	
	Parameter values	Error	Power	Error	Power	Error	Power	Error	Power	Error	Power
1.	$\delta_{1}=\delta_{2}=0$	4.8	–	4.6	–	4.8	–	4.5	–	4.5	–
2.	$\delta_{1}=0$, $\delta_{2}=0.5$	5.0	61.2	4.9	82.7	4.9	61.2	4.8	82.9	2.5	82.8
3.	$\delta_{1}=\delta_{2}=0.5$	–	94.5	–	92.3	–	94.5	–	92.2	–	92.2
4.	$\delta_{1}=\delta_{2}=\delta_{3}=0$	3.7	–	4.5	–	3.7	–	4.2	–	4.2	
5.	$\delta_{1}=\delta_{2}=0$, $\delta_{3}=0.5$	4.4	36.1	4.6	71.8	4.3	36.0	4.4	71.8	3.0	71.7
6.	$\delta_{1}=0$, $\delta_{2}=\delta_{3}=0.5$	5.0	67.3	4.6	85.6	4.8	66.8	4.4	85.4	1.6	85.4
7.	$\delta_{1}=0$, $\delta_{2}=0.25$, $\delta_{3}=0.5$	4.6	51.1	3.7	73.0	4.4	50.9	3.5	72.6	1.6	72.6
8.	$\delta_{1}=\delta_{2}=\delta_{3}=0.5$	–	93.5	–	90.7	–	93.4	–	90.4	–	90.4

The results are broadly similar to those for the fully sequential setting presented in Section 4.1. For the type I error inflator, we see that the closed *z*‐test does not control the FWER in general (as seen in scenarios 2, 6 and 7), and neither does applying the Holm procedure to the *z*‐test. The Bonferroni‐corrected *z*‐test has an inflated FWER when all null hypotheses are true, as in scenarios 1 and 4. In contrast, the adaptive tests strongly control the FWER in all scenarios. However, again this comes at the cost of reduced power. There is a slight reduction in power between the closed *z*‐test and the closed adaptive test, of between 3–4% in absolute terms. In scenarios where at least one null hypothesis is true, the Holm *z*‐test has a much higher power than the Holm adaptive test, with the power more than doubling in these scenarios, and actually tripling in scenario 6.

As for the BAR scheme, all of the methods strongly control the FWER. This time, for some scenarios the adaptive closed test basically achieves the nominal 5% level, as in scenarios 2 and 6. When there are three treatments, the Bonferroni‐corrected *z*‐test can again be overly conservative, as in scenarios 6 and 7. In contrast to the fully sequential setting, with block randomization we see that the adaptive tests actually have the highest power out of all the methods in all scenarios except scenario 2. When at least one null hypothesis is true, the Holm adaptive test has the highest power, while when all null hypotheses are false the adaptive closed test has the highest power. The power gains are small, but demonstrate that we do not always lose out in terms of power when using the proposed adaptive tests.

*Block randomization with an adaptive control allocation*: In Web Appendix F.3, we present a simulation study considering block randomization with an adaptive control allocation, as presented in Section 3.2. The results are broadly similar to those presented above.

### 4.3 Summary

In summary, the simulation results show that in the randomization settings considered, our proposed adaptive tests strongly control the FWER, as would be expected from theory. In contrast, the various *z*‐tests can all fail to control the error rate, as seen in the results for the type I error inflator. However, given a more realistic randomization scheme, such as the BAR schemes we considered, the *z*‐tests achieve strong familywise error control. As for disjunctive power, we see that when at least one null hypothesis is true, the closed tests suffer a very large drop in power compared to the Holm versions. This is because of the ‘dilution’ of the test statistic as mentioned in Section 4.1. However, when all the null hypotheses are true, then the closed test has the higher power, although the gains are at most modest.

The adaptive tests can pay a large price in terms of power when compared with the *z*‐tests, as seen in the results for the type I error inflator. In Web Appendix F.4, we give an additional simulation study with two treatments, where the randomization scheme used is simply a fixed allocation to the experimental treatments but with unequal randomization probabilities. We show that when the probability of assignment to treatment 2 is low (i.e., less than 0.2), there is a large drop in the power of the adaptive tests for testing $H_{1}$. This explains what is happening with the type I error inflator when $\delta_{1}=0$, where in the majority of trial realisations, apart from the unlikely event that treatment 1 stops early for ‘efficacy’, the probability of assignment to treatment 2 is zero by design. Hence, the type I inflator is in fact close to a worst‐case scenario for the adaptive tests. However, for adaptive randomization schemes that implement a restriction on the probability of assignment so that it is above a minimum threshold (or equivalently a minimal proportion of patients in each treatment group, we would not expect there to be a substantial loss of power when using the Holm adaptive test compared with the Holm *z*‐test, particularly in the block randomized setting.

## 5 CASE STUDY

Finally, we illustrate our proposed methodology using an example based on a phase II placebo‐controlled trial in primary hypercholesterolemia (Roth et al., 2012). The purpose of the study was to compare the effects of using the SAR236553 antibody with high‐dose or lose‐dose atorvastatin, as compared with high‐dose atorvastatin alone. The primary outcome was the least‐squares mean percent reduction from baseline of low‐density lipoprotein cholesterol (LDL‐C). Patients were randomly assigned, in a 1:1:1 ratio, to receive 80 mg of atorvastatin plus placebo, 10 mg of atorvastatin plus SAR236553, or 80 mg of atorvastatin plus SAR236553. For convenience, we label these different interventions as the ‘control’, ‘low dose’ and ‘high dose’ respectively.

In the trial, the observed least‐squares mean ± SE percent reduction from baseline in LDL‐C was 17.3±3.5 for the control, 66.2±3.5 for the low dose and 72.3±3.5 for the high dose. There were N=31 patients on the control, N=31 patients on the low dose and N=30 patients on the high dose, giving a total of N=61 patients on the two experimental doses. We use the observed values from the trial and assume that the distribution of the least‐squares standardized mean percent reduction from baseline of LDL‐C is N(17.3/3.5,1) for the control, N(66.2/3.5,1) for the low dose, and N(72.3/3.5,1) for the high dose.

Now suppose that the trial was carried out as an adaptive block randomized trial with a fixed control allocation, as described in Section 3.1. Let the trial have J=3 blocks, with block sizes (15, 15, 15) for the experimental treatments and (8, 8, 8) for the placebo. In the burn‐in period, 7 patients are allocated to the control and 8 patients are allocated to each of the experimental doses. Hence, a total of 31 patients are on the control and 61 on the experimental treatments, as in the original trial. We use the BAR scheme of Section 4.2, with priors $\mu_{i,0}=5$ and $\sigma_{i,0}^{2}=1$ (i=0,1,2), while γ=0.5.

Table 6 shows the results for a simulated trial with the above parameters, where the BAR scheme allocated 13 patients to the low dose and 32 patients to the high dose after the burn‐in period. This yields the natural weights used in the naïve *z*‐test of $n_{1}^{\prime }=21$ for the low dose and $n_{2}^{\prime }=40$ for the high dose. The natural weight for the control is $n_{0}=31$ by design. The auxiliary design randomly assigned 44 patients to the low or high dose in a 1:1 ratio, and allocated 21 patients to the low dose and 23 patients to the high dose.

**Table 6:** Test statistics, *p*‐values and weights for a simulated block randomized trial using a BAR scheme

	Low dose	High dose
*z*‐test statistic	13.76 (p<0.001)	15.50 (p<0.001)
Adaptive test statistic	12.21 (p<0.001)	16.22 (p<0.001)
Natural weights	$n_{1}^{\prime }=21$, $n_{0}=31$	$n_{2}^{\prime }=40$, $n_{0}=31$
Adaptive weights	$w^{\left(1\right)}=(30,28.05,21.49,16.43)$	$w^{\left(2\right)}=(32,34.09,42.68,46.08)$
	$w^{\left(0\right)}=(31,31.73,35.91,42.76)$	$w^{\left(0\right)}=(31,30.43,28.86,28.41)$

The adaptive test statistic is slightly smaller than the *z*‐test statistic for the low dose, while the converse is true for the test statistics for the high dose. Looking at the adaptive weights for the burn‐in period and the three blocks, we see that for the low dose, the weights for the low dose decrease for each block while the control weights increase. This pattern is reversed for the high dose. Given that all the *p*‐values are less than 0.001, using either the *z*‐test or the adaptive test we would conclude that adding the SAR236553 antibody to high‐dose or low‐dose atorvastatin leads to a statistically significant reduction in LDL‐C levels.

## 6 DISCUSSION

A major regulatory concern over the use of response‐adaptive trials in clinical practice has been ensuring control of the type I error rate. We have proposed procedures that guarantee strong familywise error control in the following multi‐armed trial settings:

1. Fully sequential response‐adaptive trials with a fixed control allocation (where the randomization rule does not depend on the control information);
2. Block‐randomized response‐adaptive trials with a fixed control allocation;
3. Block‐randomized response‐adaptive trials including an adaptive control allocation.

These procedures are applicable to a large class of RAR rules for settings (2) and (3), with only some restrictions in setting (1). Hence many Bayesian and ‘optimal’ RAR schemes proposed in the literature can be used without adjustment, with only the final test statistic having to be modified. Since our proposed adaptive testing procedures are based on the conditional invariance principle, they have the additional important flexibility of being valid when the allocation is changed due to external information (i.e., information other than the previously observed treatment assignments and patient responses). Such changes might occur due to safety data, non‐compliance or even trial protocol violations.

In practice, to control the FWER we would recommend using the Holm adaptive test. Importantly, it has a much higher power than the adaptive closed test when at least one of the null hypotheses are true. As well, it only requires *t* hypothesis tests as compared with $\left(2^{t}-1\right)$ hypothesis tests for the adaptive closed test.

Our adaptive tests lead to unequal weightings of patients, which may be controversial. Indeed, this is a more general issue that can affect the analysis of any flexible adaptive design that uses that combination test approach (Burman and Sonesson, 2006). One solution is to use the so‐called ‘dual test’, and reject a hypothesis only if both the adaptive test and the naïve *z*‐test rejects (Posch et al., 2003), but this can come at the cost of reduced power. A related problem in practice is explaining the unequal weighting of patients to clinicians, which would be a fruitful area of further investigation.

As mentioned in Section 2.4, the choice of the allocations in the auxiliary design is meant to be an approximation of the actual allocations. This choice, while not affecting the control of the FWER, can affect the power of the resulting adaptive test procedure. If information is available before the trial begins of a likely ordering of the treatment effects (such as in a trial testing different doses of a drug), then it might be possible to design ‘optimal’ auxiliary designs that maximise the expected power of the resulting adaptive test procedure.

We have assumed that the variances of the control and experimental treatments are known. Fully accounting for unknown variances would add considerable complexity to our approach. In Web Appendices F.6 and F.7, we show empirically that estimating the variance from the data (with or without assuming a common variance) does not inflate the FWER when using the Holm adaptive test, for the simulation scenarios considered in this article.

Our proposed procedures are designed for normally‐distributed outcomes but can be implemented for other types of outcomes through the use of asymptotics. For example, with survival data one could use the asymptotically standard normal form for the logrank test statistic. For binary data, a starting point would be to use the asymptotically normal test statistic for contrasting each treatment arm with the control (Jennison and Turnbull, 2000; Wason and Trippa, 2014), particularly in the block randomised setting. However, it would be useful to extend our procedures to work directly with other types of outcomes. This would be a fruitful yet challenging area for future research, since applying the conditional invariance principle in these settings without appealing to asymptotics is likely to be complex, especially given the form of the usual test statistics (such as Fisher's exact test).

As mentioned in Section 1, another possible method of controlling the FWER is through the use of (re)'randomization tests (Simon and Simon, 2011). These tests have the advantage of being valid under unknown variances, non‐normally distributed outcomes and even time trends associated with the outcome. However, our proposed adaptive testing procedures have the advantage of being valid under the composite null hypothesis $H_{i}:\delta_{i}\leq 0$ (as long as the weights for the experimental treatments are positive). Also, although it would be interesting to compare randomization tests with our proposed testing procedures, calculating the randomization *p*‐values using Monte Carlo methods for $10^{5}$ trial simulations (and repeating this for each set of parameter values) would be computationally challenging.

Finally, although we did not explicitly consider it in this article, the adaptive randomization procedures used could also incorporate covariate information, so that the allocation probabilities vary across patients with different covariates. These covariate‐adjusted RAR schemes are particularly useful when certain characteristics of the patients may be correlated with the primary outcome (Hu and Rosenberger, 2006). A related setting would be biomaker‐guided response‐adaptive trials, such as I‐SPY 2.

## Supporting information

Additional supporting information may be found online in the Supporting Information section at the end of the article.

Supplementary Materials.
