# Supplementary material for: Familywise error control in multi‐armed response‐adaptive trials
Source: Biometrics. 2019 Apr 3;75(3):885–94. doi: 10.1111/biom.13042 (PMC6739232; doi:10.1111/biom.13042)
Supplement: Supplementary file 1 — Supplementary Materials. [file BIOM-75-885-s001.pdf]

Supporting Information for "Familywise error control in multi-armed response-adaptive trials" by D.S. Robertson and J.M.S. Wason

Web Appendix A: Multi-arm response-adaptive randomization procedures

Many classes of response-adaptive randomization (RAR) schemes have been proposed in the literature, and we describe the main classes of designs below.

A.1 Urn models

A large class of RAR schemes are based on randomized urn models (Johnson and Kotz, 1977), where balls (representing the different treatments) are drawn at random to assign patients, and are replaced following some probability distribution which can depend on the observed patient outcomes. These tend to be relevant for binary and multinomial responses, because integer numbers of balls are assumed. An overview of procedures based on urn models can be found in Chapter 4 of Hu and Rosenberger (2006) and in Section 10.5 of Rosenberger and Lachin (2016).

A subclass of urn models used for multi-arm RAR is the generalized Friedman's urn (Athreya and Karlin, 1968). As special cases, this subclass includes the well-known randomized play-the winner rule (Wei and Durham, 1978) for two-arm trials, and its generalized version suitable for multi-arm clinical trials (Wei, 1979). Another subclass of urn models is known as ternary urn models, with a key example being the drop-the-loser rule by Ivanova (2003) for two-arm trials, which was generalized to multi-arm trials by Zhang et al. (2007).

A.2 Optimal allocation designs

The second large class of RAR schemes are based on optimal allocation targets, where the target allocations are based on unknown parameters of a population response model and are sequentially estimated from the accrued trial data. There are several criteria that could be chosen to optimise, and optimising can be very complicated for multi-arm trials (we refer the interested reader to Tymofyeyev et al. (2007) for optimal allocation for binary responses, and a more recent and general overview by Sverdlov and Rosenberger (2013), which covers both binary and continuous responses). Once an optimal allocation target has been identified, randomization to the different treatments proceeds via sequential estimation of the parameter values of interest. There are a number of types of sequential methods, which we list below (with a fuller overview given in Section 10.5 of Rosenberger and Lachin (2016)):

1. Doubly adaptive biased coin designs (DBCD): first introduced for the two-arm setting for exponential families by Eisele (1994). This was further developed by Hu and Zhang (2004) and then Tymofyeyev et al. (2007) for multi-arm trials (which includes as special cases a number of proposed schemes for the two-arm setting, see Hu and Rosenberger (2006, Chapter 5)). Specific examples of DBCD for continuous responses include those given by Zhang and Rosenberger (2006) and Biswas et al. (2007), with a comprehensive recent overview given by Biswas and Bhattacharya (2016).

2. Sequential maximum likelihood procedures (Melfi et al., 2001; Rosenberger et al., 2001) for multi-arm trials.

3. The efficient randomized-adaptive design (ERADE) for two-arm trials (Hu et al., 2009).

A.3 Bayesian adaptive randomization

The third large class of RAR schemes are Bayesian adaptive randomization (BAR) schemes, where the randomization probabilities are recursively updated using a Bayesian model for the patient outcomes. A popular scheme in the literature is one described in Thall and Wathen (2007) for two-arm trials, which uses a weighted posterior probability of treatment A being superior to treat-

ment B. This was then generalised to the multi-arm setting in Trippa et al. (2012) and Wason and Trippa (2014). An extension to the multi-arm setting using the *predictive* posterior probability was proposed by Yin et al. (2012).

A final related class of RAR schemes are multi-arm bandit models, which combine a decision-theoretic approach to optimal trial design with Bayesian updating. Multi-arm bandit-based randomization schemes have been proposed for both binary responses (Villar er al., 2015, 2015a; Williamson et al., 2017) as well as continuous responses (Smith and Villar, 2018).

A.4 The use of RAR in clinical trials

The use of RAR is a widely discussed and somewhat controversial topic in clinical trials. For binary responses, a number of comparisons (Korn and Freidlin, 2011; Berry, 2011; Thall et al., 2015; Wathen and Thall, 2017) have focused on the BAR scheme proposed by Thall and Wathen (2007). Particularly in the two-arm setting, fixed randomisation appears to be preferable to this scheme in terms of power and the number of treatment failures, except when the number of patients to be treated beyond the trial is small (as in rare diseases) or where there are large treatment differences (Lee et al., 2012; Du et al., 2015).

However, even in the two-arm setting, optimal response-adaptive schemes (i.e. those that target some formal optimality criteria) have been shown to have benefits over fixed randomisation by increasing both power and patient benefit simultaneously (Rosenberger et al., 2001; Rosenberger and Hu, 2004; Tymofyeyev et al., 2007; Bello and Sabo, 2016). In the multi-arm setting, which is the focus of the paper, adaptive randomisation can have further advantages over fixed randomization (Berry, 2011; Wason and Trippa, 2014; Hey and Kimmelman, 2015; Berry, 2015), particularly for more complex trial designs.

Response-adaptive designs also have application outside of the context of clinical trials. For example, multi-arm bandit models are used for market learning in economics (Bergemann and Vlimki, 2006) and to improve modern production systems that emphasize 'continuous improvement' (Scott, 2010). Some of the ethical concerns surrounding adaptive randomization (Hey and Kimmelman, 2015) would not apply in these contexts.

Web Appendix B: Derivation of the weights for familywise error control in fully sequential response-adaptive trials

Below is a diagrammatic representation of the assignments and observations for the auxiliary design compared to the actual design for the patients on the experimental treatments:

Actual design

$$\begin{array}{|ccc|ccccc}\hline a_1 & \cdots & a_r & a_{r+1} & a_{r+2} & \cdots & a_n \\ X_1 & \cdots & X_r & X_{r+1} & X_{r+2} & \cdots & X_n \\ \hline \multicolumn{3}{c}{B} & & & & \end{array}$$

Auxiliary design

$$\begin{array}{|ccc|cccccc}\hline b_1 & \cdots & b_r & b_{r+1} & b_{r+2} & \cdots & b_{n-1} & b_n \\ Y_1 & \cdots & Y_r & Y_{r+1} & Y_{r+2} & \cdots & Y_{n-1} & Y_n \\ \hline \multicolumn{3}{c}{B} & & & & & \end{array}$$

where $b_k = a_k$, $Y_k = X_k$ $(k = 1, \ldots, r)$ and $b_n \in I$ by design.

We now give a step-by-step proof of the following theorem, which gives the distribution of the test statistic for testing hypothesis H_I.

Theorem 1 Under H_I, the following test statistic is normally distributed with mean 0 and variance $(1/n'_I + 1/n_0)$

$$\tilde{T}_I = \sum_{k=1}^{n} \left(\mathbb{1}_{\{a_k \in I\}} \frac{X_k}{w_k^{(I)}} \right) - \sum_{j=1}^{n_0} \frac{X_{0j}}{w_{n,j}^{(0)}}$$

where

$$w_k^{(I)} = n'_I, \quad w_k^{(0)} = n_0 \qquad (k = 1, \ldots, r)$$

$$w_{r+l}^{(I)} = f(\lambda_{r+l}, \eta_{r+l}, \tilde{m}_{I,r+l}), \quad w_{r+l}^{(0)} = g(\lambda_{r+l}, w_{r+l}^{(I)}, \tilde{m}_{I,r+l}) \qquad (l = 1, \ldots, n-r)$$

$$w_{n,j}^{(0)} = F_1(w_{n-1}^{(0)}, m_{0,1}, m_{0,2}) \qquad (j = 1, \ldots, m_{0,1})$$

$$w_{n,j}^{(0)} = F_2(w_{n-1}^{(0)}, m_{0,1}, m_{0,2}) \qquad (j = m_{0,1}+1, \ldots, n_0)$$

$$\lambda_{r+l} = \frac{m_{I,r+l}}{w_{r+l-1}^{(I)}} - \frac{n_0}{w_{r+l-1}^{(0)}}, \quad \eta_{r+l} = \frac{m_{I,r+l}}{\left[w_{r+l-1}^{(I)}\right]^2} + \frac{n_0}{\left[w_{r+l-1}^{(0)}\right]^2} \qquad (l = 1, \ldots, n-r)$$

$$\tilde{m}_{I,r+l} = m_{I,r+l} + \mathbb{1}_{\{a_{r+l} \in I, b_{r+l} \notin I\}} - \mathbb{1}_{\{a_{r+l} \notin I, b_{r+l} \in I\}} \qquad (l = 1, \ldots, n-r)$$

$$f(\lambda, \eta, m) = \frac{\lambda m - \sqrt{m n_0 (n_0 \eta - \lambda^2)}}{\lambda^2 - n_0 \eta}, \quad g(\lambda, w, m) = \frac{n_0 w}{m - \lambda w}$$

$$F_1(w, m_1, m_2) = \mathbb{1}_{\{a_n \in I\}} w - \mathbb{1}_{\{a_n \notin I\}} \frac{m_1 \lambda_n + \sqrt{m_1 m_2 [\eta_n (m_1 + m_2) - \lambda_n^2]}}{\lambda_n^2 - m_2 \eta_n}$$

$$F_2(w, m_1, m_2) = \mathbb{1}_{\{a_n \in I\}} w - \mathbb{1}_{\{a_n \notin I\}} \frac{m_2 F_1(w, m_1, m_2)}{m_1 + \lambda_n F_1(w, m_1, m_2)}$$

$$m_{0,1} + m_{0,2} = n_0, \quad m_{0,1} > 0, \quad m_{0,2} > 0$$

Step 1

In step 1 we only consider the first response-adaptive allocation a_{r+1}. We view the auxiliary and actual trials as coming from a two-stage design, where the first stage for both is the burn-in period B, as shown below.

Auxiliary design (step 1)

$$\overbrace{\underbrace{\begin{array}{ccc} a_1 & \cdots & a_r \\ X_1 & \cdots & X_r \end{array}}_{B}}^{\text{Stage 1}} \Bigg| \overbrace{\begin{array}{cccc} b_{r+1} & b_{r+2} & \cdots & b_n \\ Y_{r+1} & Y_{r+2} & \cdots & Y_n \end{array}}^{\text{Stage 2}}$$

Actual design (step 1)

$$\overbrace{\underbrace{\begin{array}{ccc} a_1 & \cdots & a_r \\ X_1 & \cdots & X_r \end{array}}_{B}}^{\text{Stage 1}} \Bigg| \overbrace{\begin{array}{cccc} a_{r+1} & b_{r+2} & \cdots & b_n \\ X_{r+1} & Y_{r+2} & \cdots & Y_n \end{array}}^{\text{Modified stage 2}}$$

Given the interim data from B, we can determine the actual allocation a_{r+1}. Indeed, a_{r+1} needs to be known in order for the trial to continue. Hence the second stage for the actual design in step 1 is a data-dependent modification of the auxiliary design, where the allocation b_{r+1} is set to a_{r+1}. At this step, all other allocations for the actual design remain the same as the auxiliary design. The modification to the second stage in the actual design can only depend on data available at the end of the interim stage; that is, the burn-in period. Hence when considering a fully sequential response-adaptive scheme, we cannot adapt b_k to a_k for $k > r+1$ at this step, and can only consider the modification b_{r+1} to a_{r+1}.

Under the auxiliary two-stage design, the test statistic $T_I = T_I^{(1)} + T_I^{(2)}$ for the experimenal treatments is decomposed into two parts, where $T_I^{(1)}$ is calculated from the first stage data and $T_I^{(2)}$

is calculated from the second stage data. More explicitly,

$$T_I^{(1)} = \sum_{k=1}^{r} \left(\mathbb{1}_{\{a_k \in I\}} \frac{X_k}{n'_I} \right)$$
$$T_I^{(2)} = \sum_{k=r+1}^{n} \left(\mathbb{1}_{\{b_k \in I\}} \frac{Y_k}{n'_I} \right) - \sum_{j=1}^{n_0} \frac{X_{0j}}{n_0}.$$

Since the control data are independent of the design adaptations, we can consider these data as coming from the second stage of the actual and auxiliary designs.

Using the conditional invariance principle, we seek a statistic $\tilde{T}_I^{(2)}$ from the second stage data of the actual design so that under H_I and conditional on the interim data, the statistics $T_I^{(2)}$ and $\tilde{T}_I^{(2)}$ have identical conditional distributions. Hence the modified statistic $\tilde{T}_I = T_I^{(1)} + \tilde{T}_I^{(2)}$ can be used as the test statistic in the actual design, since $\tilde{T}_I$ has the same unconditional distribution under H_I.

We now select weights $w_{r+1}^{(I)}$ and $w_{r+1}^{(0)}$ so that under H_I, the statistic

$$\tilde{T}_I^{(2)} = \mathbb{1}_{\{a_{r+1} \in I\}} \frac{X_{r+1}}{w_{r+1}^{(I)}} + \sum_{k=r+2}^{n} \left(\mathbb{1}_{\{b_k \in I\}} \frac{Y_k}{w_{r+1}^{(I)}} \right) - \sum_{j=1}^{n_0} \frac{X_{0j}}{w_{r+1}^{(0)}}$$

has the same distribution as $T_I^{(2)}$ conditional on the interim data $\mathcal{D}_r^{(1)}$. Under H_I, we have

$$T_I^{(2)} \mid \mathcal{D}_r^{(1)} \sim N\left(\mu \frac{m_{I,r+1}}{n'_I} - \mu \,, \frac{m_{I,r+1}}{(n'_I)^2} + \frac{1}{n_0} \right)$$
$$\tilde{T}_I^{(2)} \mid \mathcal{D}_r^{(1)} \sim N\left(\mu \frac{\tilde{m}_{I,r+1}}{w_{r+1}^{(I)}} - \mu \frac{n_0}{w_{r+1}^{(0)}} \,, \frac{\tilde{m}_{I,r+1}}{\left(w_{r+1}^{(I)}\right)^2} + \frac{n_0}{\left(w_{r+1}^{(0)}\right)^2} \right)$$

where $\tilde{m}_{I,r+1} = m_{I,r+1} + \mathbb{1}_{\{a_{r+1} \in I, b_{r+1} \notin I\}} - \mathbb{1}_{\{a_{r+1} \notin I, b_{r+1} \in I\}}$. Here $\tilde{m}_{I,r+1}$ represents the number of allocations to treatments in I during the second stage (i.e. for patients $r+1, \ldots, n$) of step 1 of the actual design.

To match the conditional distributions we equate the conditional means and variances to give

$$w_{r+1}^{(I)} = \frac{\lambda_{r+1} \tilde{m}_{I,r+1} - \sqrt{n_0 \tilde{m}_{I,r+1} [\eta_{r+1}(n_0 + \tilde{m}_{I,r+1}) - \lambda_{r+1}^2]}}{\lambda_{r+1}^2 - n_0 \eta_{r+1}}$$
$$w_{r+1}^{(0)} = \frac{n_0 w_{r+1}^{(I)}}{\tilde{m}_{I,r+1} - \lambda_{r+1} w_{r+1}^{(I)}}$$

where $\lambda_{r+1} = m_{I,r+1}/n'_I - 1$ and $\eta_{r+1} = m_{I,r+1}/(n'_I)^2 + 1/n_0$.

Hence the full modified statistic for the actual design in step 1 is

$$\tilde{T}_{I,r+1} = \tilde{T}_I = \sum_{k=1}^{r+1} \left(\mathbb{1}_{\{a_k \in I\}} \frac{X_k}{w_k^{(I)}} \right) + \sum_{k=r+2}^{n} \left(\mathbb{1}_{\{b_k \in I\}} \frac{Y_k}{w_{r+1}^{(I)}} \right) - \sum_{j=1}^{n_0} \frac{X_{0j}}{w_{r+1}^{(0)}}$$

where we define $w_k^{(I)} = n'_I$ $(k = 1, \ldots, r)$, and the $(r+1)$ subscript on $\tilde{T}_{I,r+1}$ indicates that this is the modified test statistic for the actual design after the first $(r+1)$ patients. By the conditional invariance principle, $\tilde{T}_{I,r+1}$ is a valid test statistic for the actual design.

Step 2

In step 2, we take the actual design from step 1 as the new auxiliary design. This means that the modified test statistic $\tilde{T}_{I,r+1}$, as defined above, is also taken forward from step 1 and is the valid test statistic for the new auxiliary design. We again view the auxiliary and actual trials as two-stage designs, where this time the first stage is the data from the first $(r+1)$ patients, as shown below.

Auxiliary design (step 2)

$$\overbrace{\underbrace{\boxed{\begin{array}{ccc} a_1 & \cdots & a_r \\ X_1 & \cdots & X_r \end{array}}}_{B}\;\begin{array}{c} a_{r+1} \\ X_{r+1} \end{array}}^{\text{Stage 1}}\;\Bigg|\;\overbrace{\begin{array}{cccc} b_{r+2} & b_{r+3} & \cdots & b_n \\ Y_{r+2} & Y_{r+3} & \cdots & Y_n \end{array}}^{\text{Stage 2}}$$

Actual design (step 2)

$$\overbrace{\underbrace{\boxed{\begin{array}{ccc} a_1 & \cdots & a_r \\ X_1 & \cdots & X_r \end{array}}}_{B}\;\begin{array}{c} a_{r+1} \\ X_{r+1} \end{array}}^{\text{Stage 1}}\;\Bigg|\;\overbrace{\begin{array}{cccc} a_{r+2} & b_{r+3} & \cdots & b_n \\ X_{r+2} & Y_{r+3} & \cdots & Y_n \end{array}}^{\text{Modified stage 2}}$$

Here the second stage for the new actual design is a modification of the new auxiliary design where the allocation b_{r+2} is set to a_{r+2}.

Under the auxiliary two-stage design, the test statistic $\tilde{T}_{I,r+1}$ for the experimental treatments is decomposed into the statistics calculated from the first and second stage data, $\tilde{T}_{I,r+1} = \tilde{T}_{I,r+1}^{(1)} + \tilde{T}_{I,r+1}^{(2)}$, where now

$$\tilde{T}_{I,r+1}^{(1)} = \sum_{k=1}^{r+1} \left(\mathbb{1}_{\{a_k \in I\}} \frac{X_k}{w_k^{(I)}} \right)$$

$$\tilde{T}^{(2)}_{I,r+1} = \sum_{k=r+2}^{n} \left(\mathbb{1}_{\{b_k \in I\}} \frac{Y_k}{w^{(I)}_{r+1}} \right) - \sum_{j=1}^{n_0} \frac{X_{0j}}{w^{(0)}_{r+1}}.$$

Following the conditional invariance principle like in step 1, we seek weights $w^{(I)}_{r+2}$ and $w^{(0)}_{r+2}$ so that under H_I, the statistic

$$\tilde{T}^{(2)}_{I,r+2} = \mathbb{1}_{\{a_{r+2} \in I\}} \frac{X_{r+2}}{w^{(I)}_{r+2}} + \sum_{k=r+3}^{n} \left(\mathbb{1}_{\{b_k \in I\}} \frac{Y_k}{w^{(I)}_{r+2}} \right) - \sum_{j=1}^{n_0} \frac{X_{0j}}{w^{(0)}_{r+2}}$$

has the same distribution as $\tilde{T}^{(2)}_{I,r+1}$ conditional on the interim data $\mathcal{D}^{(1)}_{r+1}$. Matching the conditional distributions by equating the conditional means and variances gives

$$w^{(I)}_{r+2} = \frac{\lambda_{r+2}\tilde{m}_{I,r+2} - \sqrt{n_0 \tilde{m}_{I,r+2}[\eta_{r+2}(n_0 + \tilde{m}_{I,r+2}) - \lambda^2_{r+2}]}}{\lambda^2_{r+2} - n_0 \eta_{r+2}}$$
$$w^{(0)}_{r+2} = \frac{n_0 w^{(I)}_{r+2}}{\tilde{m}_{I,r+2} - \lambda_{r+2} w^{(I)}_{r+2}}$$

where

$$\lambda_{r+2} = \frac{m_{I,r+2}}{w^{(I)}_1} - \frac{n_0}{w^{(0)}_1}, \quad \eta_{r+2} = \frac{m_{I,r+2}}{\left(w^{(I)}_1\right)^2} + \frac{n_0}{\left(w^{(0)}_1\right)^2}$$

$$\tilde{m}_{I,r+2} = m_{I,r+2} + \mathbb{1}_{\{a_{r+2} \in I, b_{r+2} \notin I\}} - \mathbb{1}_{\{a_{r+2} \notin I, b_{r+2} \in I\}}.$$

Hence the full modified statistic for the actual design in step 2 is

$$\tilde{T}_{I,r+2} = \sum_{k=1}^{r+2} \left(\mathbb{1}_{\{a_k \in I\}} \frac{X_k}{w^{(I)}_k} \right) + \sum_{k=r+3}^{n} \left(\mathbb{1}_{\{b_k \in I\}} \frac{Y_k}{w^{(I)}_{r+2}} \right) - \sum_{j=1}^{n_0} \frac{X_{0j}}{w^{(0)}_{r+2}}$$

This statistic is taken forward to the next step of the process as the valid test statistic for the new auxiliary design.

Inductive step

We now repeat the process above, at each step taking forward the actual design as the new auxiliary design. The actual design at step l of the process ($l = 2, \ldots, n - r - 1$) is a modification of the

new auxiliary design where b_{r+l} is set to a_{r+l}. The valid test statistic for the new auxiliary design is $\tilde{T}_{I,r+l-1}$, taken forward from the previous step of the process, where we provide an explicit expression for the test statistics shortly. The diagrammatic representation of step l of the process is given below.

Auxiliary design (step l)

$$\overbrace{\underbrace{\boxed{\begin{array}{ccc} a_1 & \cdots & a_r \\ X_1 & \cdots & X_r \end{array}}}_{B} \begin{array}{cccc} a_{r+1} & a_{r+2} & \cdots & a_{r+l-1} \\ X_{r+1} & X_{r+2} & \cdots & X_{r+l-1} \end{array}}^{\text{Stage 1}} \Bigg| \overbrace{\begin{array}{cccc} b_{r+l} & b_{r+l+1} & \cdots & b_n \\ Y_{r+l} & Y_{r+l+1} & \cdots & Y_n \end{array}}^{\text{Stage 2}}$$

Actual design (step l)

$$\overbrace{\underbrace{\boxed{\begin{array}{ccc} a_1 & \cdots & a_r \\ X_1 & \cdots & X_r \end{array}}}_{B} \begin{array}{cccc} a_{r+1} & a_{r+2} & \cdots & a_{r+l-1} \\ X_{r+1} & X_{r+2} & \cdots & X_{r+l-1} \end{array}}^{\text{Stage 1}} \Bigg| \overbrace{\begin{array}{cccc} a_{r+l} & b_{r+l+1} & \cdots & b_n \\ X_{r+l} & Y_{r+l+1} & \cdots & Y_n \end{array}}^{\text{Modified stage 2}}$$

Using these auxiliary and actual designs, we select new weights $w^{(I)}_{r+l}$ and $w^{(0)}_{r+l}$ so that under H_I the conditional distributions of the second stage statistics are the same. This yields the new test statistic $\tilde{T}_{I,r+l}$ for the actual design in step l. For notational convenience, we introduce the following functions:

$$f(\lambda, \eta, m) = \frac{\lambda m - \sqrt{m n_0 (n_0 \eta - \lambda^2)}}{\lambda^2 - n_0 \eta}$$

$$g(\lambda, w, m) = \frac{n_0 w}{m - \lambda w}$$

Given the weights $w^{(I)}_{r+l-1}$ and $w^{(0)}_{r+l-1}$ found in the previous step, let

$$\lambda_{r+l} = \frac{m_{I,r+l}}{w^{(I)}_{r+l-1}} - \frac{n_0}{w^{(0)}_{r+l-1}}, \quad \eta_{r+l} = \frac{m_{I,r+l}}{\left(w^{(I)}_{r+l-1}\right)^2} + \frac{n_0}{\left(w^{(0)}_{r+l-1}\right)^2}$$

$$\tilde{m}_{I,r+l} = m_{I,r+l} + \mathbb{1}_{\{a_{r+l} \in I, b_{r+l} \notin I\}} - \mathbb{1}_{\{a_{r+l} \notin I, b_{r+l} \in I\}}.$$

The weights $w_{r+l}^{(I)}$ and $w_{r+l}^{(0)}$ are given by

$$\begin{aligned} w_{r+l}^{(I)} &= f(\lambda_{r+l}, \eta_{r+l}, \tilde{m}_{I,r+l}) \\ w_{r+l}^{(0)} &= g(\lambda_{r+l}, w_{r+l}^{(I)}, \tilde{m}_{I,r+l}) \end{aligned}$$

The corresponding test statistics $\tilde{T}_{I,r+k}$ for $k = 1, \dots, l$ are:

$$\tilde{T}_{I,r+k} = \sum_{j=1}^{r+k} \left(\mathbb{1}_{\{a_j \in I\}} \frac{X_j}{w_j^{(I)}} \right) + \sum_{j=r+k+1}^{n} \left(\mathbb{1}_{\{b_j \in I\}} \frac{Y_j}{w_{r+k}^{(I)}} \right) - \sum_{j=1}^{n_0} \frac{X_{0j}}{w_{r+k}^{(0)}}$$

Final step

In the final step of the process, the second stage data for the auxiliary and actual designs is a single allocation:

Auxiliary design (final step)

$$\underbrace{\begin{array}{ccc} a_1 & \cdots & a_r \\ X_1 & \cdots & X_r \end{array}}_{B} \begin{array}{cccc|c} a_{r+1} & a_{r+2} & \cdots & a_{n-1} & b_n \\ X_{r+1} & X_{r+2} & \cdots & X_{n-1} & Y_n \end{array}$$

Actual design (final step)

$$\underbrace{\begin{array}{ccc} a_1 & \cdots & a_r \\ X_1 & \cdots & X_r \end{array}}_{B} \begin{array}{cccc|c} a_{r+1} & a_{r+2} & \cdots & a_{n-1} & a_n \\ X_{r+1} & X_{r+2} & \cdots & X_{n-1} & X_n \end{array}$$

Under the auxiliary design, the test statistic $\tilde{T}_{I,n-1}$ is decomposed into the following first and second stage statistics, since $b_n \in I$ by design:

$$\tilde{T}_{I,n-1}^{(1)} = \sum_{k=1}^{n-1} \left(\mathbb{1}_{\{a_k \in I\}} \frac{X_k}{w_k^{(I)}} \right), \qquad \tilde{T}_{I,n-1}^{(2)} = \frac{Y_n}{w_{n-1}^{(I)}} - \sum_{j=1}^{n_0} \frac{X_{0j}}{w_{n-1}^{(0)}}$$

where $w_{n-1}^{(0)}$ and $w_k^{(I)}$ have been defined in the previous steps for $k = r+1, \dots, n-1$. As before, we

want to select a weight $w_n^{(I)}$ so that

$$\tilde{T}_{I,n}^{(2)} = \mathbb{1}_{\{a_n \in I\}} \frac{X_n}{w_n^{(I)}} - \sum_{j=1}^{n_0} \frac{X_{0j}}{w_n^{(0)}}$$

has the same conditional distribution as $T_{I,n-1}^{(2)}$ under H_I. If $a_n \in I$ then the auxiliary and actual designs are identical, and so $w_n^{(I)} = w_{n-1}^{(I)}$ and $w_n^{(0)} = w_{n-1}^{(0)}$.

However, if $a_n \notin I$, then we only have a single weight $w_n^{(0)}$ and hence cannot match both the conditional means and variances. Instead, we split the n_0 control observations into two groups of size $m_{0,1}$ and $m_{0,2}$, where $m_{0,1} \geq 1$, $m_{0,2} \geq 1$ and $m_{0,1} + m_{0,2} = n_0$. In practice, to keep the weights as close to the natural weight n_0 for as many of the control observations as possible, we recommend setting $m_{0,1} = n_0 - 1$ and $m_{0,2} = 1$, which is what we use for the simulation studies in Section 4.1 of the paper.

We select weights $w_{n,1}^{(0)}$ and $w_{n,2}^{(0)}$ so that under H_I, the statistic

$$\tilde{T}_{I,n}^{(2)} = -\sum_{j=1}^{m_{0,1}} \frac{X_{0j}}{w_{n,1}^{(0)}} - \sum_{j=m_{0,1}+1}^{n_0} \frac{X_{0j}}{w_{n,2}^{(0)}}$$

has the same distribution as $T_{I,n}^{(2)}$ conditional on the interim data $\mathcal{D}_{n-1}^{(1)}$. Under H_I, we have

$$T_{I,n}^{(2)} | \mathcal{D}_{n-1}^{(1)} \sim N\left(\mu \frac{1}{w_{n-1}^{(I)}} - \mu \frac{n_0}{w_{n-1}^{(0)}}, \frac{1}{\left(w_{n-1}^{(I)}\right)^2} + \frac{n_0}{\left(w_{n-1}^{(0)}\right)^2} \right)$$

$$\tilde{T}_{I,n}^{(2)} | \mathcal{D}_{n-1}^{(1)} \sim N\left(-\mu \frac{m_{0,1}}{w_{n,1}^{(0)}} - \mu \frac{m_{0,2}}{w_{n,2}^{(0)}}, \frac{m_{0,1}}{\left(w_{1,n}^{(0)}\right)^2} + \frac{m_{0,2}}{\left(w_{n,2}^{(0)}\right)^2} \right)$$

Equating the conditional means and variances gives

$$w_{n,1}^{(0)} = \frac{-m_{0,1}\lambda_n - \sqrt{m_{0,1} m_{0,2} [\eta_n (m_{0,1} + m_{0,2}) - \lambda_n^2]}}{\lambda_n^2 - m_{0,2}\eta_n}$$

$$w_{n,2}^{(0)} = -\frac{m_{0,2} w_{n,1}^{(0)}}{m_{0,1} + \lambda_n w_{n,1}^{(0)}}$$

where $\lambda_n = 1/w_{n-1}^{(I)} - n_0/w_{n-1}^{(0)}$ and $\eta_n = 1/(w_{n-1}^{(I)})^2 + n_0/(w_{n-1}^{(0)})^2$.

For notational convenience, define the following functions

$$F_1(w, m_1, m_2) = \begin{cases} w & \text{if } a_n \in I \\ \dfrac{-m_1\lambda_n - \sqrt{m_1 m_2(\eta_n(m_1+m_2) - \lambda_n^2)}}{\lambda_n^2 - m_2\eta_n} & \text{if } a_n \notin I \end{cases}$$

$$F_2(w, m_1, m_2) = \begin{cases} w & \text{if } a_n \in I \\ -\dfrac{m_2 F_1(w, m_1, m_2)}{m_1 + \lambda_n F_1(w, m_1, m_2)} & \text{if } a_n \notin I \end{cases}$$

We can express the weights $w_{n,1}^{(0)}$ and $w_{n,2}^{(0)}$ for the controls, for either $a_n \in I$ or $a_n \notin I$, as $w_{n,1}^{(0)} = F_1(w_{n-1}^{(0)}, m_{0,1}, m_{0,2})$ and $w_{n,2}^{(0)} = F_2(w_{n-1}^{(0)}, m_{0,1}, m_{0,2})$.

The final test statistic for testing hypothesis H_I is as follows:

$$\tilde{T}_I = \sum_{k=1}^{n} \left(\mathbb{1}_{\{a_k \in I\}} \frac{X_k}{w_k^{(I)}} \right) - \sum_{j=1}^{n_0} \frac{X_{0j}}{w_{n,j}^{(0)}}$$

where

$$\begin{aligned} w_k^{(I)} &= n_I', \quad w_k^{(0)} = n_0 && (k = 1, \ldots, r) \\ w_{r+l}^{(I)} &= f(\lambda_{r+l}, \eta_{r+l}, \tilde{m}_{I,r+l}) && (l = 1, \ldots, n-r) \\ w_{r+l}^{(0)} &= g(\lambda_{r+l}, w_{r+l}^{(I)}, \tilde{m}_{I,r+l}) && (l = 1, \ldots, n-r) \\ w_{n,j}^{(0)} &= F_1(w_{n-1}^{(0)}, m_{0,1}, m_{0,2}) && (j = 1, \ldots, m_{0,1}) \\ w_{n,j}^{(0)} &= F_2(w_{n-1}^{(0)}, m_{0,1}, m_{0,2}) && (j = m_{0,1}+1, \ldots, n_0) \end{aligned}$$

We reject H_I if $\tilde{T}_I$ is greater than $z_\alpha \left(1/n_I' + 1/n_0\right)^{1/2}$.

Web Appendix C: Numerical example for fully sequential response-adaptive trials

As a simple illustration of how the weights change over the course of a trial, consider the set-up given in Section 2.5 of the main paper. Below are the weights $w^{(I)}$, $w_1^{(0)}$ and $w_{10}^{(0)}$ for a variety of further actual allocations.

Table 1: *An actual allocation a that is the opposite of the auxiliary design b. The weights that would be used in the naïve z-test are* $n_0 = 10$, $n_1 = 6$ *and* $n_2 = 5$.

$a =$	1	2	1	2	1	1	2	2	1	2	1	
$b =$	1	2	2	1	2	2	1	1	2	1	*	
$w^{(1)} =$	6	6	6.81	5.83	6.81	8	6.51	4.94	6.51	4.09	4.09	$w_1^{(0)} = w_{10}^{(0)} = 10.17$
$w^{(2)} =$	6	6	5.16	6	4.94	3.81	4.94	6.51	4.10	6.51	-	$w_1^{(0)} = 9.23, w_{10}^{(0)} = -7.59$

Table 2: *An extreme actual allocation a that is equal to 1 after the burn-in period. The weights that would be used in the naïve z-test are* $n_0 = 10$, $n_1 = 10$ *and* $n_2 = 2$.

$a =$	1	2	1	1	1	1	1	1	1	1	1	
$b =$	1	2	2	1	2	2	1	1	2	1	*	
$w^{(1)} =$	6	6	6.81	6.81	8.00	9.45	9.45	9.45	12.95	12.95	12.95	$w_1^{(0)} = w_{10}^{(0)} = 8.82$
$w^{(2)} =$	6	6	5.16	5.16	4.28	3.33	3.33	3.33	2.23	2.23	-	$w_1^{(0)} = 14.73, w_{10}^{(0)} = -2.25$

Table 3: *An extreme actual allocation a that is equal to 2 after the burn-in period. The weights that would be used in the naïve z-test are* $n_0 = 10$, $n_1 = 1$ *and* $n_2 = 10$.

$a =$	1	2	2	2	2	2	2	2	2	2	2	
$b =$	1	2	2	1	2	2	1	1	2	1	*	
$w^{(1)} =$	6	6	6	5.16	5.16	5.16	4.28	3.33	3.33	2.23	-	$w_1^{(0)} = 14.73, w_{10}^{(0)} = -2.25$
$w^{(2)} =$	6	6	6	7.01	7.01	7.01	9.44	12.91	12.91	22.89	22.89	$w_1^{(0)} = w_{10}^{(0)} = 9.01$

Web Appendix D: Derivation of the weights for familywise error control in block randomized response-adaptive trials with a fixed control allocation

Defining $d_0 = 0$, let $D_l = \sum_{j=0}^{l}(r + d_j)$ for $l = 0, \ldots, J$, which represents the total number of allocations by the end of lth block, with the zeroth block corresponding to the burn-in period. For notational convenience, we let $D_{-1} = 0$. In the jth block, d_{0j} patients are allocated to the control. We define $d_{00} = 0$ and let $D_{0,l} = \sum_{j=0}^{l}(r_0 + d_{0j})$ $(l = 0, \ldots, J)$, which represents the total number of allocations to the control by the end of lth block, where $D_{0,-1} = 0$ for notational convenience. Also let $\tilde{m}_{i,J} = \sum_{k=D_J+1}^{n} \mathbb{1}_{\{a_k=i\}}$ denote the total number of allocations to the ith treatment for patients in the final block J, and define $\tilde{m}_{I,j} = \sum_{i \in I} \tilde{m}_{i,j}$.

The auxiliary design for an intersection hypothesis H_I is the same as described in Section 2.4 in the paper, except that we now impose a block structure on the auxiliary assignments to the experimental treatments. As before, the auxiliary and actual designs are identical during the burn-in period B, and we require $b_n \in I$. Below is a diagrammatic representation of the assignments and observations for the auxiliary design compared to the actual design (for the patients on the experimental treatments):

Actual design

$$\begin{array}{ccccc} \boldsymbol{a}_B & \boldsymbol{a}_1 & \boldsymbol{a}_2 & \cdots & \boldsymbol{a}_J \\ \boldsymbol{X}_B & \boldsymbol{X}_1 & \boldsymbol{X}_2 & \cdots & \boldsymbol{X}_J \end{array}$$

Auxiliary design

$$\begin{array}{ccccc} \boldsymbol{b}_B & \boldsymbol{b}_1 & \boldsymbol{b}_2 & \cdots & \boldsymbol{b}_J \\ \boldsymbol{Y}_B & \boldsymbol{Y}_1 & \boldsymbol{Y}_2 & \cdots & \boldsymbol{Y}_J \end{array}$$

Here $\boldsymbol{a}_B = (a_1, \ldots, a_r)$ and $\boldsymbol{X}_B = (X_1, \ldots, X_r)$ refer to the burn-in period B, while $\boldsymbol{a}_j = (a_{D_{j-1}+1}, \ldots, a_{D_j})$ and $\boldsymbol{X}_j = (X_{D_{j-1}+1}, \ldots, X_{D_j})$ represent the response-adaptive allocations and observations in block j $(j = 1, \ldots, J)$. By design, $\boldsymbol{b}_B = \boldsymbol{a}_B$, $\boldsymbol{Y}_B = \boldsymbol{X}_B$, while $\boldsymbol{b}_j = (b_{D_{j-1}+1}, \ldots, b_{D_j})$ and $\boldsymbol{Y}_j = (Y_{D_{j-1}+1}, \ldots, Y_{D_j})$ represent the auxiliary allocations and observations in block j $(j = 1, \ldots, J)$. As before, we require $b_n \in I$.

For the auxiliary design, let n'_i denote the total number of allocations to the ith treatment

$(i = 1, \ldots, t)$, including the burn-in period. Also let

$$m_{0,j} = \sum_{k=j}^{J} d_{0k}, \qquad m_{i,j} = \sum_{k=D_j+1}^{n} \mathbb{1}_{\{b_k=i\}}$$

denote the total number of allocations to the control and ith treatment respectively for patients in blocks $(j, j+1, \ldots, J)$. We define $n'_I = \sum_{i \in I} n'_i$ and $m_{I,j} = \sum_{i \in I} m_{i,j}$.

We now give a step-by-step proof of the following theorem, which gives the distribution of the test statistic for testing hypothesis H_I.

Theorem 2 If $\tilde{m}_{I,J} > 0$, then under H_I, the following test statistics is normally distributed with mean 0 and variance $(1/n'_I + 1/n_0)$

$$\tilde{T}_I = \sum_{j=0}^{J} \sum_{k=D_{j-1}+1}^{D_j} \mathbb{1}_{\{a_k \in I\}} \frac{X_k}{w_j^{(I)}} - \sum_{j=0}^{J} \sum_{k=D_{0,j-1}+1}^{D_{0,j}} \frac{X_{0k}}{w_j^{(0)}}$$

where

$$w_0^{(I)} = n'_I\,, \quad w_0^{(0)} = n_0$$

$$\lambda_l = \frac{m_{I,l}}{w_{l-1}^{(I)}} - \frac{m_{0,l}}{w_{l-1}^{(0)}} \qquad \text{for } l = 1, \ldots, J$$

$$\eta_l = \frac{m_{I,l}}{\left(w_{l-1}^{(I)}\right)^2} + \frac{m_{0,l}}{\left(w_{l-1}^{(0)}\right)^2} \qquad \text{for } l = 1, \ldots, J$$

$$\tilde{m}_{I,l} = m_{I,l+1} + \sum_{k=D_{l-1}+1}^{D_l} \mathbb{1}_{\{a_k \in I\}} \qquad \text{for } l = 1, \ldots, J-1$$

$$\tilde{m}_{I,J} = \sum_{k=D_{J-1}+1}^{n} \mathbb{1}_{\{a_k \in I\}}$$

$$w_j^{(I)} = f(\lambda_j, \eta_j, \tilde{m}_{I,j}, m_{0,j}) \qquad \text{for } j = 1, \ldots, J$$

$$w_j^{(0)} = g(\lambda_j, w_j^{(I)}, \tilde{m}_{I,j}, m_{0,j}) \qquad \text{for } j = 1, \ldots, J$$

$$f(\lambda, \eta, m_I, m_0) = \frac{\lambda m_I - \sqrt{m_I m_0(\eta(m_0 + m_I) - \lambda^2)}}{\lambda^2 - m_0 \eta}$$

$$g(\lambda, w, m_I, m_0) = \frac{m_0 w}{m_I - \lambda w}$$

If $\tilde{m}_{I,J} = 0$, then let $n_{0,J,1} + n_{0,J,2} = d_{0J}$, where $n_{0,J,1} > 0$, $n_{0,J,2} > 0$. Under H_I, the following

test statistics is normally distributed with mean 0 and variance $(1/n'_I + 1/n_0)$

$$\tilde{T}_I = \sum_{j=0}^{J-1} \sum_{k=D_{j-1}+1}^{D_j} \mathbb{1}_{\{a_k \in I\}} \frac{X_k}{w_j^{(I)}} - \sum_{j=0}^{J-1} \sum_{k=D_{0,j-1}+1}^{D_{0,j}} \frac{X_{0k}}{w_j^{(0)}} - \sum_{k=D_{0,J-1}+1}^{D_{0,J,1}} \frac{X_{0k}}{w_{J,1}^{(0)}} - \sum_{k=D_{0,J,1}+1}^{n_0} \frac{X_{0k}}{w_{J,2}^{(0)}}$$

where

$$w_{J,1}^{(I)} = \frac{-n_{0,J,1}\lambda_J - \sqrt{n_{0,J,1}n_{0,J,2}\left[\eta_J(n_{0,J,1}+n_{0,J,2}) - \lambda_J^2\right]}}{\lambda_J^2 - n_{0,J,2}\eta_J}$$

$$w_{J,2}^{(I)} = -\frac{n_{0,J,2}w_{J,1}^{(I)}}{n_{0,J,1} + \lambda_J w_{J,1}^{(I)}}$$

$$D_{0,J,1} = D_{0,J-1} + n_{0,J,1}$$

Step 1

In step 1 we only consider the response-adaptive allocations for the first block $\boldsymbol{a}_1$. We view the auxiliary and actual trials as coming from a two-stage design, where the first stage for both is the burn-in period B, as shown below.

Auxiliary design (step 1)

$$\begin{array}{c|cccc} & \multicolumn{4}{c}{\overbrace{\hphantom{\boldsymbol{b}_1 \quad \boldsymbol{b}_2 \quad \cdots \quad \boldsymbol{b}_J}}^{\text{Stage 2}}} \\ \boldsymbol{a}_B & \boldsymbol{b}_1 & \boldsymbol{b}_2 & \cdots & \boldsymbol{b}_J \\ \boldsymbol{X}_B & \boldsymbol{X}_1 & \boldsymbol{X}_2 & \cdots & \boldsymbol{X}_J \end{array}$$

Actual design (step 1)

$$\begin{array}{c|cccc} & \multicolumn{4}{c}{\overbrace{\hphantom{\boldsymbol{a}_1 \quad \boldsymbol{b}_2 \quad \cdots \quad \boldsymbol{b}_J}}^{\text{Modified stage 2}}} \\ \boldsymbol{a}_B & \boldsymbol{a}_1 & \boldsymbol{b}_2 & \cdots & \boldsymbol{b}_J \\ \boldsymbol{X}_B & \boldsymbol{X}_1 & \boldsymbol{Y}_2 & \cdots & \boldsymbol{Y}_J \end{array}$$

Given the interim data from B, we can determine the actual allocations $\boldsymbol{a}_1$ for the first block. Hence the second stage for the actual design in step 1 is a data-dependent modification of the auxiliary design, where the allocations $\boldsymbol{b}_1$ are set to $\boldsymbol{a}_1$. All the other allocations for the actual design remain the same as the auxiliary design.

Under the auxiliary two-stage design, the test statistic T_I is decomposed into two parts, with $T_I = T_I^{(1)} + T_I^{(2)}$, where $T_I^{(1)}$ is calculated from the first stage data and $T_I^{(2)}$ is calculated from the

second stage data. More explicitly,

$$T_I^{(1)} = \sum_{k=1}^{r} \left(\mathbb{1}_{\{a_k \in I\}} \frac{X_k}{n_I'} \right) - \sum_{k=1}^{r_0} \frac{X_{0k}}{n_0}$$
$$T_I^{(2)} = \sum_{k=r+1}^{n} \left(\mathbb{1}_{\{b_k \in I\}} \frac{Y_k}{n_I'} \right) - \sum_{k=r_0+1}^{n_0} \frac{X_{0k}}{n_0}$$

Following the conditional invariance principle, we select weights $w_1^{(I)}$ and $w_1^{(0)}$ so that under H_I, the statistic

$$\tilde{T}_I^{(2)} = \sum_{k=r+1}^{D_1} \mathbb{1}_{\{a_k \in I\}} \frac{X_k}{w_1^{(I)}} + \sum_{k=D_1+1}^{n} \mathbb{1}_{\{b_k \in I\}} \frac{Y_k}{w_1^{(I)}} - \sum_{k=r_0+1}^{n_0} \frac{X_{0k}}{w_1^{(0)}}$$

has the same distribution as $T_I^{(2)}$ conditional on the interim data $\mathcal{D}^{(1)}$. Under H_I, we have

$$T_I^{(2)} | \mathcal{D}^{(1)} \sim N\left(\mu \frac{m_{I,1}}{n_I'} - \mu \frac{m_{0,1}}{n_0'}, \frac{m_{I,1}}{(n_I')^2} + \frac{m_{0,1}}{(n_0')^2} \right)$$
$$\tilde{T}_I^{(2)} | \mathcal{D}^{(1)} \sim N\left(\mu \frac{\tilde{m}_{I,1}}{w_1^{(I)}} - \mu \frac{m_{0,1}}{w_1^{(0)}}, \frac{\tilde{m}_{I,1}}{\left(w_1^{(I)}\right)^2} + \frac{m_{0,1}}{\left(w_1^{(0)}\right)^2} \right)$$

where $\tilde{m}_{I,1} = m_{I,2} + \sum_{k=r+1}^{D_1} \mathbb{1}_{\{a_k \in I\}}$.

To match the conditional distributions we equate the conditional means and variances to give

$$w_1^{(I)} = \frac{\lambda_1 \tilde{m}_{I,1} - \sqrt{\tilde{m}_{I,1} m_{0,1} [\eta_1 (m_{0,1} + \tilde{m}_{I,1}) - \lambda_1^2]}}{\lambda_1^2 - m_{0,1} \eta_1}$$
$$w_1^{(0)} = \frac{m_{0,1} w_1^{(I)}}{\tilde{m}_{I,1} - \lambda_1 w_1^{(I)}}$$

where $\lambda_1 = m_{I,1}/n_I' - m_{0,1}/n_0$ and $\eta_1 = m_{I,1}/(n_I')^2 + m_{0,1}/(n_0)^2$.

Hence the full modified statistic for the actual design in step 1 is

$$\tilde{T}_{I,1} = \tilde{T}_I = \sum_{j=0}^{1} \sum_{k=D_{j-1}+1}^{D_j} \mathbb{1}_{\{a_k \in I\}} \frac{X_k}{w_j^{(I)}} + \sum_{k=D_1+1}^{n} \mathbb{1}_{\{b_k \in I\}} \frac{Y_k}{w_1^{(I)}} - \sum_{k=1}^{r_0} \frac{X_{0k}}{w_0^{(0)}} - \sum_{k=r_0+1}^{n_0} \frac{X_{0k}}{w_1^{(0)}}$$

where $w_0^{(0)} = n_0$ and $w_0^{(I)} = n_I'$. This statistic is taken forward to the next step of the process as the valid test statistic for the new auxiliary design.

Inductive step

We continue the process above, at each step taking forward the actual design as the new auxiliary design. The actual design at step l of the process ($l \in \{1, \dots, J-1\}$) is a modification of the new auxiliary design where the allocations $\boldsymbol{b}_l$ are set to $\boldsymbol{a}_l$. The valid test statistic for the new auxiliary design is $\tilde{T}_{I,l}$, taken forward from the previous step of the process. The diagrammatic representation of step l of the process is given below:

Auxiliary design (step l)

$$
\begin{array}{cccc|cccc}
\multicolumn{4}{c|}{\overbrace{\hphantom{\boldsymbol{a}_B \quad \boldsymbol{a}_1 \quad \cdots \quad \boldsymbol{a}_{l-1}}}^{\text{Stage 1}}} & \multicolumn{4}{c}{\overbrace{\hphantom{\boldsymbol{b}_l \quad \boldsymbol{b}_{l+1} \quad \cdots \quad \boldsymbol{b}_J}}^{\text{Stage 2}}} \\
\boldsymbol{a}_B & \boldsymbol{a}_1 & \cdots & \boldsymbol{a}_{l-1} & \boldsymbol{b}_l & \boldsymbol{b}_{l+1} & \cdots & \boldsymbol{b}_J \\
\boldsymbol{X}_B & \boldsymbol{X}_1 & \cdots & \boldsymbol{X}_{l-1} & \boldsymbol{Y}_l & \boldsymbol{Y}_{l+1} & \cdots & \boldsymbol{Y}_J
\end{array}
$$

Actual design (step l)

$$
\begin{array}{cccc|cccc}
\multicolumn{4}{c|}{\overbrace{\hphantom{\boldsymbol{a}_B \quad \boldsymbol{a}_1 \quad \cdots \quad \boldsymbol{a}_{l-1}}}^{\text{Stage 1}}} & \multicolumn{4}{c}{\overbrace{\hphantom{\boldsymbol{a}_l \quad \boldsymbol{b}_{l+1} \quad \cdots \quad \boldsymbol{b}_J}}^{\text{Modified stage 2}}} \\
\boldsymbol{a}_B & \boldsymbol{a}_1 & \cdots & \boldsymbol{a}_{l-1} & \boldsymbol{a}_l & \boldsymbol{b}_{l+1} & \cdots & \boldsymbol{b}_J \\
\boldsymbol{X}_B & \boldsymbol{X}_1 & \cdots & \boldsymbol{X}_{l-1} & \boldsymbol{X}_l & \boldsymbol{Y}_{l+1} & \cdots & \boldsymbol{Y}_J
\end{array}
$$

Using these auxiliary and actual designs, we select new weights $w_l^{(I)}$ and $w_l^{(0)}$ so that under H_I the conditional distributions of the second stage statistics are the same. This yields the new test statistic $\tilde{T}_{I,l}$ for the actual design in step l. For notational convenience, we introduce the following functions:

$$
\begin{aligned}
f(\lambda, \eta, m_I, m_0) &= \frac{\lambda m_I - \sqrt{m_I m_0[\eta(m_0 + m_I) - \lambda^2]}}{\lambda^2 - m_0 \eta} \\
g(\lambda, w, m_I, m_0) &= \frac{m_0 w}{m_I - \lambda w}
\end{aligned}
$$

Given the weights $w_{l-1}^{(I)}$ and $w_{l-1}^{(0)}$ found in the previous step, let

$$
\lambda_l = \frac{m_{I,l}}{w_{l-1}^{(I)}} - \frac{m_{0,l}}{w_{l-1}^{(0)}}, \quad \eta_l = \frac{m_{I,l}}{\left(w_{l-1}^{(I)}\right)^2} + \frac{m_{0,l}}{\left(w_{l-1}^{(0)}\right)^2}
$$

$$
\tilde{m}_{I,l} = m_{I,l+1} + \sum_{k=D_{l-1}+1}^{D_l} \mathbb{1}_{\{a_k \in I\}}
$$

The weights $w_l^{(I)}$ and $w_l^{(0)}$ are given by

$$w_l^{(I)} = f(\lambda_l, \eta_l, \tilde{m}_{I,l}, m_{0,l})$$
$$w_l^{(0)} = g(\lambda_l, w_l^{(I)}, \tilde{m}_{I,l}, m_{0,l})$$

The corresponding test statistic $\tilde{T}_{I,l}$ is:

$$\tilde{T}_{I,l} = \sum_{j=0}^{l} \sum_{k=D_{j-1}+1}^{D_j} \mathbb{1}_{\{a_k \in I\}} \frac{X_k}{w_j^{(I)}} + \sum_{k=D_l+1}^{n} \mathbb{1}_{\{b_k \in I\}} \frac{Y_k}{w_l^{(I)}} - \sum_{j=0}^{l} \sum_{k=D_{0,j-1}+1}^{D_{0,j}} \frac{X_{0k}}{w_j^{(0)}} - \sum_{k=D_{0,l}+1}^{n_0} \frac{X_{0k}}{w_l^{(0)}}$$

where we define $D_{0,-1} = 0$.

Final step

In the final step of the process, the second stage data for the auxiliary and actual designs is the final block:

Auxiliary design (final step)

$$\overbrace{\begin{matrix} \boldsymbol{a}_B & \boldsymbol{a}_1 & \cdots & \boldsymbol{a}_{J-1} \\ \boldsymbol{X}_B & \boldsymbol{X}_1 & \cdots & \boldsymbol{X}_{J-1} \end{matrix}}^{\text{Stage 1}} \left| \begin{matrix} \boldsymbol{b}_J \\ \boldsymbol{Y}_J \end{matrix} \right.$$

Actual design (step 2)

$$\overbrace{\begin{matrix} \boldsymbol{a}_B & \boldsymbol{a}_1 & \cdots & \boldsymbol{a}_{J-1} \\ \boldsymbol{X}_B & \boldsymbol{X}_1 & \cdots & \boldsymbol{X}_{J-1} \end{matrix}}^{\text{Stage 1}} \left| \begin{matrix} \boldsymbol{a}_J \\ \boldsymbol{X}_J \end{matrix} \right.$$

Under the auxiliary design, the test statistic $\tilde{T}_{I,J-1}$ is decomposed into the following first and second stage statistics, where $b_n \in I$ by design:

$$\tilde{T}_{I,J-1}^{(1)} = \sum_{j=0}^{J-1} \sum_{k=D_{j-1}+1}^{D_j} \mathbb{1}_{\{a_k \in I\}} \frac{X_k}{w_j^{(I)}} - \sum_{j=0}^{J-1} \sum_{k=D_{0,j-1}+1}^{D_{0,j}} \frac{X_{0k}}{w_j^{(0)}}$$

$$\tilde{T}^{(2)}_{I,J-1} = \sum_{k=D_{J-1}+1}^{n} \mathbb{1}_{\{b_k \in I\}} \frac{Y_k}{w^{(0)}_{J-1}} - \sum_{k=D_{0,J-1}+1}^{n_0} \frac{X_{0k}}{w^{(0)}_{J-1}}$$

where $w^{(0)}_j$ and $w^{(I)}_j$ have been defined in the previous steps for $j = 1, \ldots, J-1$. We want to select weights $w^{(I)}_J$ and $w^{(0)}_J$ so that

$$\tilde{T}^{(2)}_{I,J} = \sum_{k=D_{J-1}+1}^{n} \mathbb{1}_{\{a_k \in I\}} \frac{X_k}{w^{(I)}_J} - \sum_{k=D_{0,J-1}+1}^{n_0} \frac{X_{0k}}{w^{(0)}_J}$$

has the same conditional distribution as $T^{(2)}_{I,J-1}$ under H_I. Let

$$\lambda_J = \frac{m_{I,J}}{w^{(I)}_{J-1}} - \frac{m_{0,J}}{w^{(0)}_{J-1}}, \quad \eta_J = \frac{m_{I,J}}{\left(w^{(I)}_{J-1}\right)^2} + \frac{m_{0,J}}{\left(w^{(0)}_{J-1}\right)^2}$$

If $\tilde{m}_{I,J} > 0$, then the weights $w^{(I)}_J$ and $w^{(0)}_J$ are given by

$$\begin{aligned} w^{(I)}_J &= f(\lambda_J, \eta_J, \tilde{m}_{I,J}, m_{0,J}) \\ w^{(0)}_J &= g(\lambda_J, w^{(I)}_J, \tilde{m}_{I,J}, m_{0,J}) \end{aligned}$$

In this case, the final test statistic for testing hypothesis H_I is as follows:

$$\tilde{T}_I = \sum_{j=0}^{J} \sum_{k=D_{j-1}+1}^{D_j} \mathbb{1}_{\{a_k \in I\}} \frac{X_k}{w^{(I)}_j} - \sum_{j=0}^{J} \sum_{k=D_{0,j-1}+1}^{D_{0,j}} \frac{X_{0k}}{w^{(0)}_j}$$

where

$$\begin{aligned} w^{(I)}_0 &= n'_I, \quad w^{(0)}_0 = n_0 \\ w^{(I)}_j &= f(\lambda_j, \eta_j, \tilde{m}_{I,j}, m_{0,j}) \qquad (j = 1, \ldots, J) \\ w^{(0)}_j &= g(\lambda_j, w^{(I)}_j, \tilde{m}_{I,j}, m_{0,j}) \qquad (j = 1, \ldots, J) \end{aligned}$$

We reject H_I if $\tilde{T}_I$ is greater than $z_\alpha(1/n'_I + 1/n'_0)^{1/2}$.

However, if $\tilde{m}_{I,J} = 0$ then we only have a single weight $w^{(0)}_J$ and hence cannot match both the conditional means and variances. In this case, since by design $d_{0J} > 1$, we consider separately the first $n_{0,J,1}$ control observations and the next $n_{0,J,2}$ control observations, where $n_{0,J,1} > 0$, $n_{0,J,2} > 0$

and $n_{0,J,1}+n_{0,J,2}=d_{0J}$. In order to keep the weights as close to the natural weight n_0 for as many of the control observations as possible, we recommend setting $n_{0,J,1}=d_{0J}-1$ and $n_{0,J,2}=1$, which is what we use for the simulation studies in Section 4.2 of the paper.

Letting $D_{0,J,1}=D_{0,J-1}+n_{0,J,1}$, we select weights $w_{J,1}^{(0)}$ and $w_{J,2}^{(0)}$ so that under H_I, the statistic

$$\tilde{T}_{I,J}^{(2)} = -\sum_{k=D_{0,J-1}+1}^{D_{0,J,1}} \frac{X_{0k}}{w_{J,1}^{(0)}} - \sum_{k=D_{0,J,1}+1}^{n_0} \frac{X_{0k}}{w_{J,2}^{(0)}}$$

has the same distribution as $T_{I,J-1}^{(2)}$ conditional on the interim data $\mathcal{D}^{(1)}$. Under H_I, we have

$$\tilde{T}_{I,J}^{(2)}|\mathcal{D}^{(1)} \sim N\left(-\mu\frac{n_{0,J,1}}{w_{J,1}^{(0)}} - \mu\frac{n_{0,J,2}}{w_{J,2}^{(0)}}, \frac{n_{0,J,1}}{\left(w_{J,1}^{(0)}\right)^2} + \frac{n_{0,J,2}}{\left(w_{J,2}^{(0)}\right)^2}\right)$$

Equating the conditional means and variances gives

$$w_{J,1}^{(0)} = \frac{-n_{0,J,1}\lambda_J - \sqrt{n_{0,J,1}n_{0,J,2}[\eta_J(n_{0,J,1}+n_{0,J,2})-\lambda_J^2]}}{\lambda_J^2 - n_{0,J,2}\eta_J}$$
$$w_{J,2}^{(0)} = -\frac{n_{0,J,2}w_{J,1}^{(I)}}{n_{0,J,1}+\lambda_J w_{J,1}^{(I)}}$$

In this case, the final test statistic for testing hypothesis H_I is as follows:

$$\tilde{T}_I = \sum_{j=0}^{J-1}\sum_{k=D_{j-1}+1}^{D_j} \mathbb{1}_{\{a_k\in I\}}\frac{X_k}{w_j^{(I)}} - \sum_{j=0}^{J-1}\sum_{k=D_{0,j-1}+1}^{D_{0,j}} \frac{X_{0k}}{w_j^{(0)}} - \sum_{k=D_{0,J-1}+1}^{D_{0,J,1}} \frac{X_{0k}}{w_{J,1}^{(0)}} - \sum_{k=D_{0,J,1}+1}^{n_0} \frac{X_{0k}}{w_{J,2}^{(0)}}$$

We reject H_I if $\tilde{T}_I$ is greater than $z_\alpha(1/n_I'+1/n_0)^{1/2}$.

Web Appendix E: Derivation of the weights for familywise error control in block randomized response-adaptive trials with an adaptive control allocation

Let $a_k = 0$ if the kth patient is allocated to the control and $n_0 = \sum_{k=1}^{n} \mathbb{1}_{\{a_k=0\}}$ denote the total number of allocations to the control. The naïve z-test for H_I rejects H_I if the test statistic

$$T_I = \sum_{k=1}^{n} \left(\mathbb{1}_{\{a_k \in I\}} \frac{X_k}{n_I} \right) - \sum_{k=1}^{n} \left(\mathbb{1}_{\{a_k=0\}} \frac{X_k}{n_0} \right)$$

is greater than $z_\alpha \left(1/n_I + 1/n_0\right)^{1/2}$.

The trial starts with a a burn-in period B, which allocates $r_0 > 0$ patients to the control and $r_i > 0$ patients to the ith treatment ($i = 1, \dots, h$), where r_0 and the r_i are fixed in advance. Hence a total of $r = \sum_{i=0}^{t} r_i$ patients are allocated to the experimental treatments during the burn-in period. The auxiliary design for hypothesis H_I starts with a burn-in period B with r patients that is identical to the actual design. The subsequent $n - r - 2$ allocations are given by a fixed sequence $(b_{r+1}, \dots, b_{n-2})$. The allocation b_{n-1} is to the control, while the allocation b_n must be in I. For the auxiliary design, let n'_0 and n'_i denote the total number of allocations to the control and the ith treatment respectively ($i = 1, \dots, t$), including the burn-in period.

We now give a step-by-step proof of the following theorem, which gives the distribution of the test statistic for testing hypothesis H_I.

Theorem 3 If $m_{I,J} > 0$ and $m_{0,J} > 0$, then under H_I, the following test statistics is normally distributed with mean 0 and variance $(1/n'_I + 1/n'_0)$:

$$\tilde{T}_I = \sum_{j=0}^{J} \sum_{k=D_{j-1}+1}^{D_j} \mathbb{1}_{\{a_k \in I\}} \frac{X_k}{w_j^{(I)}} - \sum_{j=0}^{J} \sum_{k=D_{j-1}+1}^{D_j} \mathbb{1}_{\{a_k=0\}} \frac{X_k}{w_j^{(0)}}$$

where

$$w_0^{(I)} = n'_I\,, \quad w_0^{(0)} = n'_0$$

$$w_j^{(I)} = f(\lambda_j, \eta_j, \tilde{m}_{I,j}, \tilde{m}_{0,j}) \qquad \text{for } j = 1, \dots, J$$

$$w_j^{(0)} = g(\lambda_j, w_j^{(I)}, \tilde{m}_{I,j}, \tilde{m}_{0,j}) \qquad \text{for } j = 1, \dots, J$$

$$\tilde{m}_{I,J} = \sum_{k=D_{J-1}+1}^{n} \mathbb{1}_{\{a_k \in I\}}$$

$$\tilde{m}_{0,J} = \sum_{k=D_{J-1}+1}^{n} \mathbb{1}_{\{a_k = 0\}}$$

If $m_{I,J} = 0$ and $m_{0,J} > 1$, let $n_{0,J,1} + n_{0,J,2} = m_{0,J}$, where $n_{0,J,1} > 0$ and $n_{0,J,2} > 0$. Suppose the $(D_{0,J,1})$-th patient receives the $(n_{0,J,1})$-th allocation to the control in block J. Under H_I, the following test statistics is normally distributed with mean 0 and variance $(1/n'_I + 1/n'_0)$:

$$\begin{aligned}\tilde{T}_I = & \sum_{j=0}^{J-1} \sum_{k=D_{j-1}+1}^{D_j} \mathbb{1}_{\{a_k \in I\}} \frac{X_k}{w_j^{(I)}} - \sum_{j=0}^{J-1} \sum_{k=D_{j-1}+1}^{D_j} \mathbb{1}_{\{a_k = 0\}} \frac{X_k}{w_j^{(0)}} \\ & - \sum_{k=D_{J-1}+1}^{D_{0,J,1}} \mathbb{1}_{\{a_k = 0\}} \frac{X_k}{w_{J,1}^{(0)}} - \sum_{k=D_{0,J,1}+1}^{n} \mathbb{1}_{\{a_k = 0\}} \frac{X_k}{w_{J,2}^{(0)}}\end{aligned}$$

where

$$w_{J,1}^{(I)} = \frac{-n_{0,J,1}\lambda_J - \sqrt{n_{0,J,1}n_{0,J,2}[\eta_J(n_{0,J,1} + n_{0,J,2}) - \lambda_J^2]}}{\lambda_J^2 - n_{0,J,2}\eta_J}$$

$$w_{J,2}^{(I)} = -\frac{n_{0,J,2}w_{J,1}^{(I)}}{n_{0,J,1} + \lambda_J w_{J,1}^{(I)}}$$

If $m_{0,J} = 0$ and $m_{I,J} > 1$, then let $n_{I,J,1} + n_{I,J,2} = m_{I,J}$, where $n_{I,J,1} > 0$ and $n_{I,J,2} > 0$. Suppose the $(D_{I,J,1})$-th patient receives the $(n_{I,J,1})$-th allocation to a treatment in I in block J. Under H_I, the following test statistics is normally distributed with mean 0 and variance $(1/n'_I + 1/n'_0)$:

$$\begin{aligned}\tilde{T}_I = & \sum_{j=0}^{J-1} \sum_{k=D_{j-1}+1}^{D_j} \mathbb{1}_{\{a_k \in I\}} \frac{X_k}{w_j^{(I)}} - \sum_{j=0}^{J-1} \sum_{k=D_{j-1}+1}^{D_j} \mathbb{1}_{\{a_k = 0\}} \frac{X_k}{w_j^{(0)}} \\ & + \sum_{k=D_{J-1}+1}^{D_{I,J,1}} \mathbb{1}_{\{a_k \in I\}} \frac{X_k}{w_{J,1}^{(I)}} + \sum_{k=D_{I,J,1}+1}^{n} \mathbb{1}_{\{a_k \in I\}} \frac{X_k}{w_{J,2}^{(I)}}\end{aligned}$$

where

$$w_{J,1}^{(I)} = \frac{n_{I,J,1}\lambda_J - \sqrt{n_{I,J,1}n_{I,J,2}[\eta_J(n_{I,J,1} + n_{I,J,2}) - \lambda_J^2]}}{\lambda_J^2 - n_{I,J,2}\eta_J}$$

$$w_{J,2}^{(I)} = \frac{n_{I,J,2}w_{J,1}^{(I)}}{\lambda_J w_{J,1}^{(I)} - n_{I,J,1}}$$

Step 1

In step 1 we only consider the response-adaptive allocations for the first block $\boldsymbol{a}_1$. We view the auxiliary and actual trials as coming from a two-stage design, where the first stage for both is the burn-in period B, as shown below.

Auxiliary design (step 1)

$$\begin{array}{c|cccc} & \multicolumn{4}{c}{\overbrace{\hspace{6em}}^{\text{Stage 2}}} \\ \boldsymbol{a}_B & \boldsymbol{a}_1 & \boldsymbol{a}_2 & \cdots & \boldsymbol{a}_J \\ \boldsymbol{X}_B & \boldsymbol{X}_1 & \boldsymbol{X}_2 & \cdots & \boldsymbol{X}_J \end{array}$$

Actual design (step 1)

$$\begin{array}{c|cccc} & \multicolumn{4}{c}{\overbrace{\hspace{6em}}^{\text{Modified stage 2}}} \\ \boldsymbol{a}_B & \boldsymbol{a}_1 & \boldsymbol{b}_2 & \cdots & \boldsymbol{b}_J \\ \boldsymbol{X}_B & \boldsymbol{X}_1 & \boldsymbol{Y}_2 & \cdots & \boldsymbol{Y}_J \end{array}$$

Under the auxiliary two-stage design, the test statistic $T_I = T_I^{(1)} + T_I^{(2)}$ for the experimenal treatments is decomposed into two parts, where $T_I^{(1)}$ is calculated from the first stage data and $T_I^{(2)}$ is calculated from the second stage data. More explicitly,

$$T_I^{(1)} = \sum_{k=1}^{r}\left(\mathbb{1}_{\{a_k\in I\}}\frac{X_k}{n'_I}\right) - \sum_{k=1}^{r}\left(\mathbb{1}_{\{a_k=0\}}\frac{X_k}{n'_0}\right)$$
$$T_I^{(2)} = \sum_{k=r+1}^{n}\left(\mathbb{1}_{\{b_k\in I\}}\frac{Y_k}{n'_I}\right) - \sum_{k=r+1}^{n}\left(\mathbb{1}_{\{b_k=0\}}\frac{Y_k}{n'_0}\right)$$

We now select weights $w_1^{(I)}$ and $w_1^{(0)}$ so that under H_I, the statistic

$$\tilde{T}_I^{(2)} = \sum_{k=r+1}^{D_1}\mathbb{1}_{\{a_k\in I\}}\frac{X_k}{w_1^{(I)}} + \sum_{k=D_1+1}^{n}\mathbb{1}_{\{b_k\in I\}}\frac{Y_k}{w_1^{(I)}} - \sum_{k=r+1}^{D_1}\mathbb{1}_{\{a_k=0\}}\frac{X_k}{w_1^{(0)}} - \sum_{k=D_1+1}^{n}\mathbb{1}_{\{b_k=0\}}\frac{Y_k}{w_1^{(0)}}$$

has the same distribution as $T_I^{(2)}$ conditional on the interim data $\mathcal{D}^{(1)}$. Under H_I, we have

$$T_I^{(2)}|\mathcal{D}^{(1)} \sim N\left(\mu\frac{m_{I,1}}{n'_I} - \mu\frac{m_{0,1}}{n'_0}, \frac{m_{I,1}}{(n'_I)^2} + \frac{m_{0,1}}{(n'_0)^2}\right)$$
$$\tilde{T}_I^{(2)}|\mathcal{D}^{(1)} \sim N\left(\mu\frac{\tilde{m}_{I,1}}{w_1^{(I)}} - \mu\frac{\tilde{m}_{0,1}}{w_1^{(0)}}, \frac{\tilde{m}_{I,1}}{\left(w_1^{(I)}\right)^2} + \frac{\tilde{m}_{0,1}}{\left(w_1^{(0)}\right)^2}\right)$$

where

$$\tilde{m}_{I,1} = m_{I,2} + \sum_{k=r+1}^{D_1} \mathbb{1}_{\{a_k \in I\}}, \quad \tilde{m}_{0,1} = m_{0,2} + \sum_{k=r+1}^{D_1} \mathbb{1}_{\{a_k = 0\}}.$$

To match the conditional distributions we equate the conditional means and variances to give

$$\begin{aligned} w_1^{(I)} &= \frac{\lambda_1 \tilde{m}_{I,1} - \sqrt{\tilde{m}_{I,1}\tilde{m}_{0,1}[\eta_1(\tilde{m}_{0,1} + \tilde{m}_{I,1}) - \lambda_1^2]}}{\lambda_1^2 - \tilde{m}_{0,1}\eta_1} \\ w_1^{(0)} &= \frac{\tilde{m}_{0,1} w_1^{(I)}}{\tilde{m}_{I,1} - \lambda_1 w_1^{(I)}} \end{aligned}$$

where $\lambda_1 = m_{I,1}/n'_I - m_{0,1}/n'_0$ and $\eta_1 = m_{I,1}/(n'_I)^2 + m_{0,1}/(n'_0)^2$.

Hence the full modified statistic for the actual design in step 1 is

$$\begin{aligned} \tilde{T}_{I,1} = \tilde{T}_I = &\sum_{j=0}^{1} \sum_{k=D_{j-1}+1}^{D_j} \mathbb{1}_{\{a_k \in I\}} \frac{X_k}{w_j^{(I)}} + \sum_{k=D_1+1}^{n} \mathbb{1}_{\{b_k \in I\}} \frac{Y_k}{w_1^{(I)}} \\ &- \sum_{j=0}^{1} \sum_{k=D_{j-1}+1}^{D_j} \mathbb{1}_{\{a_k = 0\}} \frac{X_k}{w_j^{(0)}} - \sum_{k=D_1+1}^{n} \mathbb{1}_{\{b_k = 0\}} \frac{Y_k}{w_1^{(0)}} \end{aligned}$$

where we define $D_{-1} = 0$, $w_0^{(I)} = n'_I$ and $w_0^{(0)} = n'_0$. By the conditional invariance principle, $\tilde{T}_{I,1}$ is a valid test statistic for the actual design.

Inductive step

We now repeat the process above, at each step taking forward the actual design as the new auxiliary design. The actual design at step l of the process ($l \in \{1, \ldots, J-1\}$) is a modification of the new auxiliary design where the allocations $\boldsymbol{b}_l$ are set to $\boldsymbol{a}_l$. The valid test statistic for the new auxiliary design is $\tilde{T}_{I,l}$, taken forward from the previous step of the process. The diagrammatic representation of step l of the process is given below.

Auxiliary design (step l)

Stage 1				Stage 2			
$\boldsymbol{a}_B$	$\boldsymbol{a}_1$	$\cdots$	$\boldsymbol{a}_{l-1}$	$\boldsymbol{b}_l$	$\boldsymbol{b}_{l+1}$	$\cdots$	$\boldsymbol{b}_J$
$\boldsymbol{X}_B$	$\boldsymbol{X}_1$	$\cdots$	$\boldsymbol{X}_{l-1}$	$\boldsymbol{Y}_l$	$\boldsymbol{Y}_{l+1}$	$\cdots$	$\boldsymbol{Y}_J$

Actual design (step l)

$$\begin{array}{cccc|cccc} \multicolumn{4}{c|}{\overbrace{\hspace{10em}}^{\text{Stage 1}}} & \multicolumn{4}{c}{\overbrace{\hspace{10em}}^{\text{Modified stage 2}}} \\ \boldsymbol{a}_B & \boldsymbol{a}_1 & \cdots & \boldsymbol{a}_{l-1} & \boldsymbol{a}_l & \boldsymbol{b}_{l+1} & \cdots & \boldsymbol{b}_J \\ \boldsymbol{X}_B & \boldsymbol{X}_1 & \cdots & \boldsymbol{X}_{l-1} & \boldsymbol{X}_l & \boldsymbol{Y}_{l+1} & \cdots & \boldsymbol{Y}_J \end{array}$$

Using these auxiliary and actual designs, we select new weights $w_l^{(I)}$ and $w_l^{(0)}$ so that under H_I the conditional distributions of the second stage statistic are the same. This yields the new test statistic $\tilde{T}_{I,l}$ for the actual design in step l.

For notational convenience, we use the following functions as before:

$$f(\lambda, \eta, m_I, m_0) = \frac{\lambda m_I - [m_I m_0 \{\eta(m_0 + m_I) - \lambda^2\}]^{1/2}}{\lambda^2 - m_0 \eta}$$
$$g(\lambda, w, m_I, m_0) = \frac{m_0 w}{m_I - \lambda w}$$

Given the weights $w_{l-1}^{(I)}$ and $w_{l-1}^{(0)}$ found in the previous step, let

$$\lambda_l = \frac{m_{I,l}}{w_{l-1}^{(I)}} - \frac{m_{0,l}}{w_{l-1}^{(0)}}, \quad \eta_l = \frac{m_{I,l}}{\left(w_{l-1}^{(I)}\right)^2} + \frac{m_{0,l}}{\left(w_{l-1}^{(0)}\right)^2}$$

$$\tilde{m}_{I,l} = m_{I,l+1} + \sum_{k=D_{l-1}+1}^{D_l} \mathbb{1}_{\{a_k \in I\}}$$

$$\tilde{m}_{0,l} = m_{0,l+1} + \sum_{k=D_{l-1}+1}^{D_l} \mathbb{1}_{\{a_k = 0\}}$$

The weights $w_l^{(I)}$ and $w_l^{(0)}$ are given by

$$w_l^{(I)} = f(\lambda_l, \eta_l, \tilde{m}_{I,l}, \tilde{m}_{0,l})$$
$$w_l^{(0)} = g(\lambda_l, w_l^{(I)}, \tilde{m}_{I,l}, \tilde{m}_{0,l})$$

The corresponding test statistic $\tilde{T}_{I,l}$ is:

$$\begin{aligned}\tilde{T}_{I,l} = & \sum_{j=0}^{l} \sum_{k=D_{j-1}+1}^{D_j} \mathbb{1}_{\{a_k \in I\}} \frac{X_k}{w_j^{(I)}} + \sum_{k=D_l+1}^{n} \mathbb{1}_{\{b_k \in I\}} \frac{Y_k}{w_l^{(I)}} \\ & - \sum_{j=0}^{l} \sum_{k=D_{j-1}+1}^{D_j} \mathbb{1}_{\{a_k = 0\}} \frac{X_k}{w_j^{(0)}} - \sum_{k=D_l+1}^{n} \mathbb{1}_{\{b_k = 0\}} \frac{Y_k}{w_l^{(0)}}\end{aligned}$$

Final step

Under the auxiliary design, the test statistic $\tilde{T}_{I,J-1}$ is decomposed into the following first and second stage statistics, where $b_{n-1} = 0$ and $b_n \in I$ by design:

$$\begin{aligned}\tilde{T}_{I,J-1}^{(1)} &= \sum_{j=0}^{J-1} \sum_{k=D_{j-1}+1}^{D_j} \mathbb{1}_{\{a_k \in I\}} \frac{X_k}{w_j^{(I)}} - \sum_{j=0}^{J-1} \sum_{k=D_{j-1}+1}^{D_j} \mathbb{1}_{\{a_k = 0\}} \frac{X_k}{w_j^{(0)}} \\ \tilde{T}_{I,J-1}^{(2)} &= \sum_{k=D_{J-1}+1}^{n} \mathbb{1}_{\{b_k \in I\}} \frac{Y_k}{w_{J-1}^{(0)}} - \sum_{k=D_{J-1}+1}^{n} \mathbb{1}_{\{b_k = 0\}} \frac{Y_k}{w_{J-1}^{(0)}}\end{aligned}$$

where $w_j^{(0)}$ and $w_j^{(I)}$ have been defined in the previous steps for $j = 1, \dots, J-1$. We want to select weights $w_J^{(I)}$ and $w_J^{(0)}$ so that

$$\tilde{T}_{I,J}^{(2)} = \sum_{k=D_{J-1}+1}^{n} \mathbb{1}_{\{a_k \in I\}} \frac{X_k}{w_J^{(I)}} - \sum_{k=D_{J-1}+1}^{n} \mathbb{1}_{\{a_k = 0\}} \frac{X_k}{w_J^{(0)}}$$

has the same conditional distribution as $T_{I,J-1}^{(2)}$ under H_I. For notational convenience, let

$$\lambda_J = \frac{m_{I,J}}{w_{J-1}^{(I)}} - \frac{m_{0,J}}{w_{J-1}^{(0)}}, \quad \eta_J = \frac{m_{I,J}}{\left(w_{J-1}^{(I)}\right)^2} + \frac{m_{0,J}}{\left(w_{J-1}^{(0)}\right)^2}$$

If $m_{I,J} > 0$ and $m_{0,J} > 0$, then let

$$\tilde{m}_{I,J} = \sum_{k=D_{J-1}+1}^{n} \mathbb{1}_{\{a_k \in I\}}, \quad \tilde{m}_{0,J} = \sum_{k=D_{J-1}+1}^{n} \mathbb{1}_{\{a_k = 0\}}.$$

The weights $w_J^{(I)}$ and $w_J^{(0)}$ are given by

$$w_J^{(I)} = f(\lambda_J, \eta_J, \tilde{m}_{I,J}, \tilde{m}_{0,J})$$

$$w_J^{(0)} = g(\lambda_J, w_J^{(I)}, \tilde{m}_{I,J}, \tilde{m}_{0,J})$$

[Note that in the rare case where $\lambda_J^2 = \tilde{m}_{0,J}\eta_J$, then $w_J^{(I)} = \frac{\tilde{m}_{I,J}(\tilde{m}_{0,J}+\tilde{m}_{I,J})}{-2\tilde{m}_{I,J}\lambda_J}$ instead]

Hence the final test statistic for testing hypothesis H_I is as follows:

$$\tilde{T}_I = \sum_{j=0}^{J} \sum_{k=D_{j-1}+1}^{D_j} \mathbb{1}_{\{a_k \in I\}} \frac{X_k}{w_j^{(I)}} - \sum_{j=0}^{J} \sum_{k=D_{j-1}+1}^{D_j} \mathbb{1}_{\{a_k = 0\}} \frac{X_k}{w_j^{(0)}}$$

where

$$w_0^{(I)} = n_I', \quad w_0^{(0)} = n_0'$$

$$w_j^{(I)} = f(\lambda_j, \eta_j, \tilde{m}_{I,j}, \tilde{m}_{0,j}) \qquad (j = 1, \ldots, J)$$

$$w_j^{(0)} = g(\lambda_j, w_j^{(I)}, \tilde{m}_{I,j}, \tilde{m}_{0,j}) \qquad (j = 1, \ldots, J)$$

We reject H_I if $\tilde{T}_I$ is greater than $z_\alpha \left(1/n_I' + 1/n_0'\right)^{1/2}$.

However, if $m_{I,J} = 0$ and $m_{0,J} > 1$, then we only have a single weight $w_J^{(0)}$ and hence cannot match both the conditional means and variances. In this case, we consider separately the first $n_{0,J,1}$ control observations and the next $n_{0,J,2}$ control observations, where $n_{0,J,1} > 0$, $n_{0,J,2} > 0$ and $n_{0,J,1} + n_{0,J,2} = m_{0,J}$. As before, we recommend setting $n_{0,J,1} = m_{0,J} - 1$ and $n_{0,J,2} = 1$, which is what we use for the simulation studies in Section F.3.

Suppose the $(D_{0,J,1})$th patient receives the $(n_{0,J,1})$th allocation to the control in block J. We select weights $w_{J,1}^{(0)}$ and $w_{J,2}^{(0)}$ so that under H_I, the statistic

$$\tilde{T}_{I,J}^{(2)} = -\sum_{k=D_{J-1}+1}^{D_{0,J,1}} \mathbb{1}_{\{a_k = 0\}} \frac{X_k}{w_{J,1}^{(0)}} - \sum_{k=D_{0,J,1}+1}^{n} \mathbb{1}_{\{a_k = 0\}} \frac{X_k}{w_{J,2}^{(0)}}$$

has the same distribution as $T_{I,J-1}^{(2)}$ conditional on the interim data $\mathcal{D}^{(1)}$. Under H_I, we have

$$\tilde{T}_{I,J}^{(2)} | \mathcal{D}^{(1)} \sim N\left(-\mu \frac{n_{0,J,1}}{w_{J,1}^{(0)}} - \mu \frac{n_{0,J,2}}{w_{J,2}^{(0)}}, \frac{n_{0,J,1}}{\left(w_{J,1}^{(0)}\right)^2} + \frac{n_{0,J,2}}{\left(w_{J,2}^{(0)}\right)^2} \right)$$

Equating the conditional means and variances gives

$$w_{J,1}^{(0)} = \frac{-n_{0,J,1}\lambda_J - \sqrt{n_{0,J,1}n_{0,J,2}[\eta_J(n_{0,J,1}+n_{0,J,2}) - \lambda_J^2]}}{\lambda_J^2 - n_{0,J,2}\eta_J}$$

$$w_{J,2}^{(0)} = -\frac{n_{0,J,2}w_{J,1}^{(I)}}{n_{0,J,1} + \lambda_J w_{J,1}^{(I)}}$$

In this case, the final test statistic for testing hypothesis H_I is as follows:

$$\tilde{T}_I = \sum_{j=0}^{J-1} \sum_{k=D_{j-1}+1}^{D_j} \mathbb{1}_{\{a_k \in I\}} \frac{X_k}{w_j^{(I)}} - \sum_{j=0}^{J-1} \sum_{k=D_{j-1}+1}^{D_j} \mathbb{1}_{\{a_k = 0\}} \frac{X_k}{w_j^{(0)}} - \sum_{k=D_{J-1}+1}^{D_{0,J,1}} \mathbb{1}_{\{a_k=0\}} \frac{X_k}{w_{J,1}^{(0)}} - \sum_{k=D_{0,J,1}+1}^{n} \mathbb{1}_{\{a_k=0\}} \frac{X_k}{w_{J,2}^{(0)}}$$

We reject H_I if $\tilde{T}_I$ is greater than $z_\alpha \left(1/n_I' + 1/n_0'\right)^{1/2}$.

If $m_{0,J} = 0$ and $m_{I,J} > 1$, then we only have a single weight $w_J^{(I)}$ and hence cannot match both the conditional means and variances. In this case, we consider separately the first $n_{I,J,1}$ and the next $n_{I,J,2}$ observations from treatments in I, where $n_{I,J,1} > 0$, $n_{I,J,2} > 0$ and $n_{I,J,1} + n_{I,J,2} = m_{I,J}$. We recommend setting $n_{I,J,1} = m_{I,J} - 1$ and $n_{I,J,2} = 1$, which is used for the simulation studies in Section F.3.

Suppose the $(D_{I,J,1})$th patient receives the $(n_{I,J,1})$th allocation to a treatment in I in block J. We select weights $w_{J,1}^{(I)}$ and $w_{J,2}^{(I)}$ so that under H_I, the statistic

$$\tilde{T}_{I,J}^{(2)} = \sum_{k=D_{J-1}+1}^{D_{I,J,1}} \mathbb{1}_{\{a_k \in I\}} \frac{X_k}{w_{J,1}^{(I)}} + \sum_{k=D_{I,J,1}+1}^{n} \mathbb{1}_{\{a_k \in I\}} \frac{X_k}{w_{J,2}^{(I)}}$$

has the same distribution as $T_{I,J-1}^{(2)}$ conditional on the interim data $\mathcal{D}^{(1)}$. Under H_I, we have

$$T_{I,J-1}^{(2)} | \mathcal{D}^{(1)} \sim N\left(\mu \frac{m_{I,J}}{w_{J-1}^{(I)}} - \mu \frac{m_{0,J}}{w_{J-1}^{(0)}}, \frac{m_{I,J}}{\left(w_{J-1}^{(I)}\right)^2} + \frac{m_{0,J}}{\left(w_{J-1}^{(0)}\right)^2}\right)$$

$$\tilde{T}_{I,J}^{(2)} | \mathcal{D}^{(1)} \sim N\left(\mu \frac{n_{I,J,1}}{w_{J,1}^{(I)}} + \mu \frac{n_{I,J,2}}{w_{J,2}^{(I)}}, \frac{n_{I,J,1}}{\left(w_{J,1}^{(I)}\right)^2} + \frac{n_{I,J,2}}{\left(w_{J,2}^{(I)}\right)^2}\right)$$

Equating the conditional means and variances gives

$$w_{J,1}^{(I)} = \frac{n_{I,J,1}\lambda_J - \sqrt{n_{I,J,1}n_{I,J,2}[\eta_J(n_{I,J,1}+n_{I,J,2}) - \lambda_J^2]}}{\lambda_J^2 - n_{I,J,2}\eta_J}$$
$$w_{J,2}^{(I)} = \frac{n_{I,J,2}w_{J,1}^{(I)}}{\lambda_J w_{J,1}^{(I)} - n_{I,J,1}}$$

In this case, the final test statistic for testing hypothesis H_I is as follows:

$$\tilde{T}_I = \sum_{j=0}^{J-1}\sum_{k=D_{j-1}+1}^{D_j} \mathbb{1}_{\{a_k \in I\}} \frac{X_k}{w_j^{(I)}} - \sum_{j=0}^{J-1}\sum_{k=D_{j-1}+1}^{D_j} \mathbb{1}_{\{a_k = 0\}} \frac{X_k}{w_j^{(0)}} + \sum_{k=D_{J-1}+1}^{D_{I,J,1}} \mathbb{1}_{\{a_k \in I\}} \frac{X_k}{w_{J,1}^{(I)}} + \sum_{k=D_{I,J,1}+1}^{n} \mathbb{1}_{\{a_k \in I\}} \frac{X_k}{w_{J,2}^{(I)}}$$

We reject H_I if $\tilde{T}_I$ is greater than $z_\alpha\left(1/n'_I + 1/n'_0\right)^{1/2}$.

If $\max(m_{0,J}, m_{I,J}) \leq 1$ and $\min(m_{0,J}, m_{I,J}) = 0$ then we cannot match the conditional means and variances and hence our adaptive procedure fails. However, such a scenario is unlikely given reasonably large block sizes and a minimum allocation probability to the control, for example. In our simulation study in Section F.3, this scenario was never observed. To investigate this further, let p_0 and p_I denote the probability of assigning a patient in the final block to the control and a treatment $i \in I$, respectively. Recalling that the final block is of size d_J, then the probability p^* of $\max(m_{0,J}, m_{I,J}) \leq 1$ and $\min(m_{0,J}, m_{I,J}) = 0$ is as follows:

$$\begin{aligned} p^* &= \text{pr}(m_{0,J} = 0, m_{I,J} = 0) + \text{pr}(m_{0,J} = 0, m_{I,J} = 1) + \text{pr}(m_{0,J} = 1, m_{I,J} = 0) \\ &= (1 - p_0 - p_I)^{d_J} + d_J p_I (1 - p_0 - p_I)^{d_J - 1} + d_J p_0 (1 - p_0 - p_I)^{d_J - 1} \end{aligned}$$

Hence as long as d_J is large and p_0 (or p_I) are not too small, then p^* will be close to zero. As an example, if there is a minimum allocation probability of 0.2 to the control (so that $p_0 \geq 0.2$) then $p^* < 0.01$ for $d_J > 31$.

Finally, it is possible that the weights for the final block are not real-valued and hence the procedure fails to give a valid test statistic. When we do have real weights for the final block, it can also happen that the weights for the experimental treatment are negative. In this case, our procedure

no longer necessarily controls the FWER for the composite null hypotheses $H_i : \delta_i \leq 0$, but only the point null hypotheses $H_i : \delta_i = 0$. Hence the adaptive test that allows for response-adaptive allocation to the control does so at the cost of being less robust and flexible. Web Appendix F.3 gives some simulation results to illustrate these two issues.

Web Appendix F: Additional simulation results

F.1 Fully sequential randomization

Type I error inflator: For $t = 2$ treatments, this is the same randomization scheme as presented in Section 2.3 of the paper. For $t = 3$ treatments, if $\sum_{j=1}^{k}(\mathbb{1}_{\{a_j=1\}}X_j/n_{1k}) > 0.5$, then we randomize patient $(k+1)$ to treatments 2 and 3 with equal probability.

BAR: The efficacy outcome for the ith experimental treatment follows a $N(\mu_i, 1)$ distribution. For simplicity, we assign independent normal priors to the μ_i, so that $\mu_i \sim N(\mu_{i,0}, \sigma_{i,0}^2)$, and let $n_{i,K} = \sum_{k=1}^{K} \mathbb{1}_{\{a_k=i\}}$. After observing the efficacy outcomes $\boldsymbol{x} = (x_1, \ldots, x_K)$ for the first K patients, the posterior for μ_i is as follows:

$$\mu_i \mid \boldsymbol{X} = \boldsymbol{x} \sim N\left(\frac{\sigma_{i,0}^2}{1 + n_{i,K}\sigma_{i,0}^2}\sum_{k=1}^{K}\mathbb{1}_{\{a_k=i\}}x_k + \frac{n_{i,K}}{1 + n_{i,K}\sigma_{i,0}^2}\mu_{i,0}\,,\ \frac{\sigma_{i,0}^2}{1 + n_{i,K}\sigma_{i,0}^2}\right)$$

We use a suggested BAR scheme of Yin et al. (2012). For $t = 2$ experimental treatments, the randomization probabilities $(\pi_1, 1 - \pi_1)$ after observing the Kth patient are:

$$\pi_1 = \frac{P(\mu_1 > \mu_2 \mid \boldsymbol{X} = \boldsymbol{x})^\tau}{P(\mu_1 > \mu_2 \mid \boldsymbol{X} = \boldsymbol{x})^\tau + \{1 - P(\mu_1 > \mu_2 \mid \boldsymbol{X} = \boldsymbol{x})\}^\tau}$$

For $t > 2$ experimental treatments, we first obtain the average of the posterior means $\bar{\mu} = \frac{1}{t}\sum_{i=1}^{t}\mu_i$. The randomization probabilities π_i after observing the Kth patient are:

$$\pi_i = \frac{P(\mu_i > \bar{\mu} \mid \boldsymbol{X} = \boldsymbol{x})^\tau}{\sum_{j=1}^{t} P(\mu_j > \bar{\mu} \mid \boldsymbol{X} = \boldsymbol{x})^\tau}$$

In our simulations, for simplicity we set the priors $\mu_{i,0} = 0$ and $\sigma_{i,0}^2 = 1$, while $\tau = 0.5$.

F.2 Block randomization with a fixed control allocation

Type I error inflator: The allocation probabilities for block $j \in \{1, \ldots, J-1\}$, patient $k = D_j + 1, \ldots, D_{j+1}$ and treatment $l \in \{2, \ldots, t\}$ are:

$$P(a_k = 1) = \begin{cases} 0 & \text{if } \sum_{i=1}^{D_j} \mathbb{1}_{\{a_i=1\}} \frac{X_i}{n_{1,j}} > 0.5 \\ 1 & \text{otherwise} \end{cases}$$

$$P(a_k = l) = \begin{cases} 1/(t-1) & \text{if } \sum_{i=1}^{D_j} \mathbb{1}_{\{a_i=1\}} \frac{X_i}{n_{1,j}} > 0.5 \\ 0 & \text{otherwise} \end{cases}$$

where $n_{1,j} = \sum_{i=1}^{D_j} \mathbb{1}_{\{a_i=1\}}$.

BAR: The efficacy outcome for the ith treatment follows a $N(\mu_i, 1)$ distribution. For notational convenience, let $\mu_0 = \mu$; that is, the mean of the control. We assign independent normal priors to the μ_i ($i = 0, 1, \ldots, t$), such that $\mu_i \sim N(\mu_{i,0}, \sigma^2_{i,0})$. At stage $(j+1)$, when the efficacy outcomes $\boldsymbol{x} = (x_1, \ldots, x_{D_j})$ have been observed, the posterior for μ_i is as follows:

$$\mu_i \mid \boldsymbol{X} = \boldsymbol{x} \sim N\left(\frac{\sigma^2_{i,0}}{1 + n_{i,K}\sigma^2_{i,0}} \sum_{k=1}^{D_j} \mathbb{1}_{\{a_k=i\}} x_k + \frac{n_{i,K}}{1 + n_{i,K}\sigma^2_{i,0}} \mu_{i,0} \, , \frac{\sigma^2_{i,0}}{1 + n_{i,K}\sigma^2_{i,0}} \right)$$

where $n_{i,K} = \sum_{k=1}^{D_K} \mathbb{1}_{\{a_k=i\}}$.

We use a similar BAR scheme to the one in Wason and Trippa (2014). If there are t experimental treatments, the randomization probabilities $(\pi_1, \ldots, \pi_h)$ for the experimental treatments at the $(j+1)$th stage are:

$$\pi_i = \frac{P(\mu_i > \mu_0 \mid \boldsymbol{X} = \boldsymbol{x})^\gamma}{\sum_{l=1}^{t} P(\mu_l > \mu_0 \mid \boldsymbol{X} = \boldsymbol{x})^\gamma}$$

In our simulations, for simplicity we set the priors $\mu_{i,0} = 0$ and $\sigma^2_{i,0} = 1$, while $\gamma = 0.5$.

F.3 Block randomization with an adaptive allocation to the control

We consider block randomization with an adaptive control allocation, as briefly introduced in Section 3.3 of the paper. We use the setup of a trial with $J = 3$ blocks and sizes (50, 50, 50). In the

burn-in period, 5 patients are allocated to each of the treatments including the control. We again set the true control mean $\mu = 0$, and $\alpha = 0.05$.

Type I error inflator: The allocation probabilities for block $j \in \{1, \dots, J-1\}$, patient $k = D_j + 1, \dots, D_{j+1}$ and treatment $l \in \{0, 2, \dots, t\}$ are:

$$P(a_k = 1) = \begin{cases} 0 & \text{if } \sum_{i=1}^{D_j} \mathbb{1}_{\{a_i=1\}} \frac{X_i}{n_{1,j}} > 0.5 \\ 1 & \text{otherwise} \end{cases}$$

$$P(a_k = l) = \begin{cases} 1/t & \text{if } \sum_{i=1}^{D_j} \mathbb{1}_{\{a_i=1\}} \frac{X_i}{n_{1,j}} > 0.5 \\ 0 & \text{otherwise} \end{cases}$$

where $n_{1,j} = \sum_{i=1}^{D_j} \mathbb{1}_{\{a_i=1\}}$.

Bayesian adaptive randomization: The priors and posteriors are the same as in Section 4.3 in the paper, and we use a similar Bayesian adaptive randomization scheme. If there are t experimental treatments, then the randomization probabilities $(\pi_0, \pi_1, \dots, \pi_h)$ at the $(j+1)$th stage are:

$$\pi_i \propto \begin{cases} \dfrac{P(\mu_i > \mu_0 \mid X_1 = x_1, \dots, X_{D_j} = x_{D_j})^\gamma}{\sum_{l=1}^{t} P(\mu_l > \mu_0 \mid X_1 = x_1, \dots, X_{D_j} = x_{D_j})^\gamma} & (i = 1, \dots, h) \\ \frac{1}{t} \exp\left(\max(\hat{m}_{1j}, \hat{m}_{2j}, \dots, \hat{m}_{hj}) - \hat{m}_{0j}\right)^\nu & (i = 0) \end{cases}$$

where $\hat{m}_{ij}$ is the current arm-specific sample size for the ith treatment at the end of the jth stage. In our simulations, for simplicity we set the priors $\mu_{i,0} = 0$ and $\sigma_{i,0}^2 = 1$, while $\gamma = 0.5$ and $\nu = 0.1$.

Simulation results: Table 4 gives the results for the type I error inflator randomization scheme, while Table 5 gives the result for BAR. For each scenario, we ran 10^5 simulated trials. The auxiliary designs in all scenarios were random draws from a discrete uniform distribution on $\{0, 1, \dots, t\}$.

The results here are again broadly similar to those for the fully sequential setting, and the block randomization setting with a fixed allocation to the control. For the type I error inflator, the various z-tests do not strongly control the FWER. The adaptive tests do achieve strong error control, but this comes at the cost of a very large decrease in power when compared with the Holm z-test.

For the BAR scheme, again all methods strongly control the FWER. This time, the z-tests have

Table 4: *Familywise error rate and disjunctive power for the type I error inflator, for block randomization with an adaptive control allocation. There were 10^5 simulated trials for each set of parameter values.*

	Adaptive closed test		Adaptive test (Holm)		Closed z-test		z-test (Holm)		z-test (Bonferroni)	
Parameter values	Error	Power	Error	Power	Error	Power	Error	Power	Error	Power
1. $\delta_1 = \delta_2 = 0$	3.8	-	4.9	-	4.4	-	**6.7**	-	**6.7**	-
2. $\delta_1 = 0$, $\delta_2 = 1$	4.8	19.1	3.7	25.1	**8.2**	25.4	**7.8**	67.2	4.3	67.1
3. $\delta_1 = \delta_2 = 0.5$	-	90.1	-	84.4	-	93.3	-	89.9	-	89.9
4. $\delta_1 = \delta_2 = \delta_3 = 0$	3.2	-	4.1	-	3.9	-	**6.2**	-	**6.2**	-
5. $\delta_1 = \delta_2 = 0$, $\delta_3 = 1$	3.8	14.0	4.4	21.9	4.8	20.0	**6.5**	61.7	4.8	61.6
6. $\delta_1 = 0$, $\delta_2 = \delta_3 = 1$	4.8	19.1	3.4	24.6	**8.4**	26.4	**7.5**	80.6	3.2	80.6
7. $\delta_1 = 0$, $\delta_2 = 0.5$, $\delta_3 = 1$	4.5	17.0	3.0	22.6	**8.0**	23.7	**6.6**	66.9	2.9	66.8
8. $\delta_1 = \delta_2 = \delta_3 = 0.5$	-	87.5	-	78.4	-	91.8	-	86.9	-	86.9

Table 5: *Familywise error rate and disjunctive power for BAR, for block randomization with an adaptive control allocation. There were 10^5 simulated trials for each set of parameter values.*

	Adaptive closed test		Adaptive test (Holm)		Closed z-test		z-test (Holm)		z-test (Bonferroni)	
Parameter values	Error	Power	Error	Power	Error	Power	Error	Power	Error	Power
1. $\delta_1 = \delta_2 = 0$	4.6	-	4.5	-	4.6	-	4.4	-	4.4	-
2. $\delta_1 = 0$, $\delta_2 = 0.5$	5.0	54.4	4.9	76.4	4.8	56.1	4.7	78.0	2.4	78.0
3. $\delta_1 = \delta_2 = 0.5$	-	90.6	-	87.4	-	91.5	-	88.3	-	88.3
4. $\delta_1 = \delta_2 = \delta_3 = 0$	4.0	-	4.3	-	3.9	-	4.2	-	4.2	-
5. $\delta_1 = \delta_2 = 0$, $\delta_3 = 0.5$	4.6	29.0	4.5	61.4	4.6	29.9	4.4	62.9	3.1	62.9
6. $\delta_1 = 0$, $\delta_2 = \delta_3 = 0.5$	4.9	56.4	4.5	76.1	4.7	57.4	4.4	77.3	1.6	77.3
7. $\delta_1 = 0$, $\delta_2 = 0.25$, $\delta_3 = 0.5$	4.5	41.7	3.6	62.9	4.3	42.7	3.5	63.7	1.7	63.7
8. $\delta_1 = \delta_2 = \delta_3 = 0.5$	-	85.9	-	82.0	-	86.9	-	83.0	-	83.0

the highest power. When at least one null hypothesis is true the Holm z-test has the highest power, although there is only a small gain compared to the Holm adaptive test. When all null hypotheses are false, the closed z-test has a slightly higher power than the closed adaptive test.

With an adaptive control allocation, the weights of the adaptive test can become imaginary, or negative for the experimental treatments. In the former case, we set the modified test statistics $\tilde{T}_I = -\infty$ and do not reject the null hypothesis H_I, which will preserve the FWER at the cost of lower power. In the latter case, we cannot use the adaptive test for the composite null hypotheses $H_i : \delta_i \leq 0$, although it will still be a valid test for the point null hypotheses $H_i : \delta_i = 0$.

We also considered how often at least one imaginary or negative weight occurs over the 10^5

simulations for the two randomization schemes. Tables 6 and 7 give the percentage of simulations where the weights for the experimental treatments are imaginary or negative, for the type I error inflator and BAR scheme respectively.

Table 6: *Percentage of simulations where at least one imaginary or negative weight occurs for the type I error inflator, with* 10^5 *simulated trials for each set of parameter values.*

	Adaptive closed test		Adaptive test (Holm)	
Parameter values	Imaginary	Negative	Imaginary	Negative
1. $\delta_1 = \delta_2 = 0$	0.09	0.00	0.09	0.00
2. $\delta_1 = 0$, $\delta_2 = 1$	0.09	0.00	0.09	0.00
3. $\delta_1 = \delta_2 = 0.5$	0.07	0.00	0.07	0.00
4. $\delta_1 = \delta_2 = \delta_3 = 0$	6.81	0.00	0.18	0.00
5. $\delta_1 = \delta_2 = 0$, $\delta_3 = 1$	6.86	0.00	0.17	0.00
6. $\delta_1 = 0$, $\delta_2 = \delta_3 = 1$	6.84	0.00	0.17	0.00
7. $\delta_1 = 0$, $\delta_2 = 0.5$, $\delta_3 = 1$	6.89	0.00	0.17	0.00
8. $\delta_1 = \delta_2 = \delta_3 = 0.5$	2.67	0.00	0.53	0.00

Table 7: *Percentage of simulations where at least one imaginary or negative weight occurs for BAR, with* 10^5 *simulated trials for each set of parameter values.*

	Adaptive closed test		Adaptive test (Holm)	
Parameter values	Imaginary	Negative	Imaginary	Negative
1. $\delta_1 = \delta_2 = 0$	0.10	0.09	0.10	0.09
2. $\delta_1 = 0$, $\delta_2 = 1$	0.14	0.10	0.14	0.10
3. $\delta_1 = \delta_2 = 0.5$	0.00	0.08	0.00	0.08
4. $\delta_1 = \delta_2 = \delta_3 = 0$	0.29	0.12	0.29	0.12
5. $\delta_1 = \delta_2 = 0$, $\delta_3 = 1$	0.35	0.07	0.34	0.07
6. $\delta_1 = 0$, $\delta_2 = \delta_3 = 1$	0.25	0.08	0.25	0.08
7. $\delta_1 = 0$, $\delta_2 = 0.5$, $\delta_3 = 1$	0.20	0.11	0.19	0.11
8. $\delta_1 = \delta_2 = \delta_3 = 0.5$	0.01	0.08	0.01	0.08

For the type I error inflator, there are essentially no negative weights for either adaptive test. The Holm adaptive tests has imaginary weights less than than 0.6% of the time in all scenarios. In contrast, when there are three treatments, the adaptive closed tests can have up to 7% of the simulations having imaginary weights. This shows that for more extreme randomization schemes, the adaptive closed test is not very robust, and is much less robust than the Holm adaptive test. For BAR, the percentage of imaginary weights is less than 0.4% for either adaptive test. This time there are negative weights in some simulations, but the percentage is very low, at less than 0.2%.

F.4 Power of the adaptive test

The adaptive tests can pay a large price in terms of power when compared with the z-tests, as seen in the results for the type I error inflator. In order to understand what is happening in this setting, we conducted an additional simulation study. Suppose we are testing $t = 2$ treatments, and that the randomization scheme used is simply a fixed allocation to the experimental treatments, but with unequal randomization probabilities. Let p_2 denote the probability of assignment to treatment 2.

Firstly consider the fully sequential trial setup of Section 4.1 in the paper, with $\delta_1 = 0$, $\delta_2 = 0.7$. Figure 1 shows how the power of the Holm adaptive test and z-test compares as p_2 varies. We see that when $p_2 > 0.5$, the adaptive test only suffers a small loss of power compared to the z-test. However, when $p_2 < 0.5$, the adaptive test loses an increasing amount of power.

Now consider the block randomization setup of Section 4.2 in the paper, with $\delta_1 = 0$, $\delta_2 = 0.5$. Figure 2 shows that this time, the power of the adaptive test is very close, or even equal, to the z-test when $p_2 > 1/3$. This shows how the adaptive test is more robust in terms of power in the block randomization setting compared to the fully sequential version.

Figure 3 shows how the powers differ for $\delta_1 = 0$, $\delta_2 = 1$ and $p_2 < 0.2$. We can see that when $p_2 < 0.15$, there is a noticeable and increasing divergence between the powers of the two tests. Indeed, when $p_2 = 0$ the power of the Holm z-test is three times that of the Holm adaptive test. This shows what is happening with the type I error inflator when $\delta_1 = 0$, where in the majority of trial scenarios, apart from the unlikely event that treatment 1 stops early for 'efficacy', $p_2 = 0$ by design. Hence, the type I inflator is in fact close to a worst-case scenario for the adaptive tests.

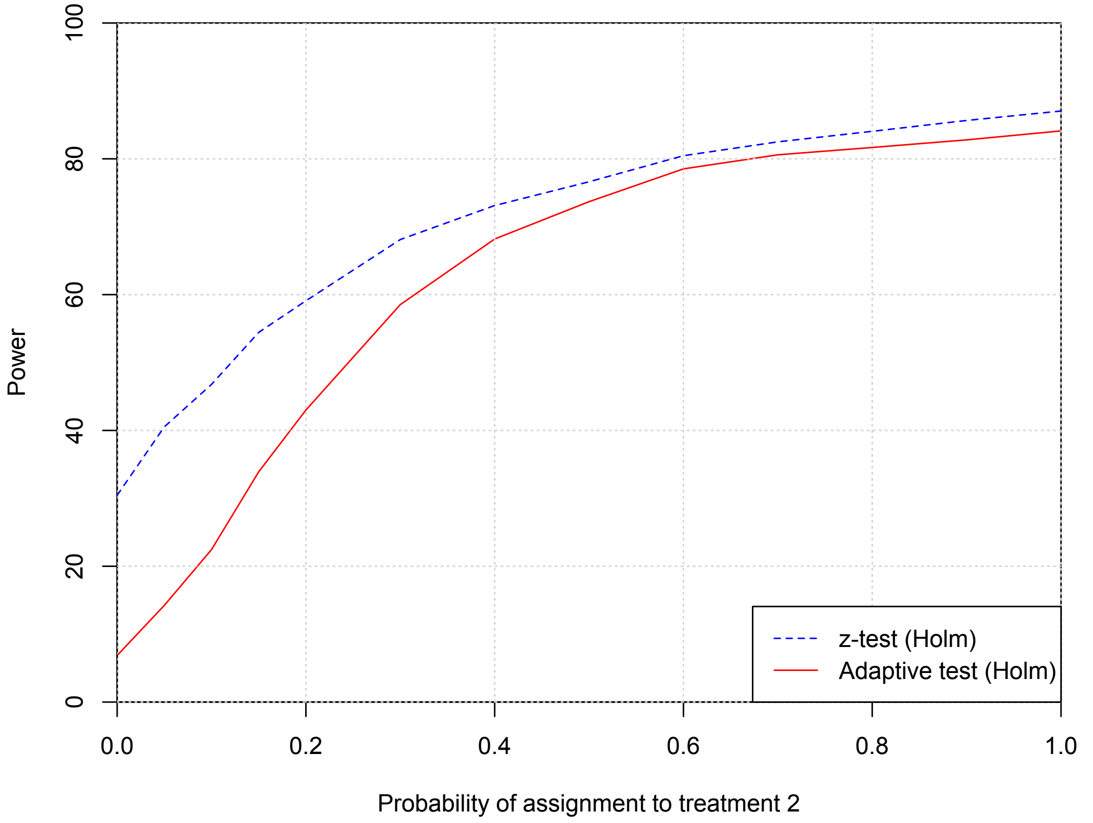

Figure 1: *Power of the Holm adaptive test and Holm* z*-test as a function of the probability of assignment to treatment 2. We use the fully sequential trial setup of Section 4.2 in the paper, with* $t = 2$ *treatments and* $\delta_1 = 0$, $\delta_2 = 0.7$.

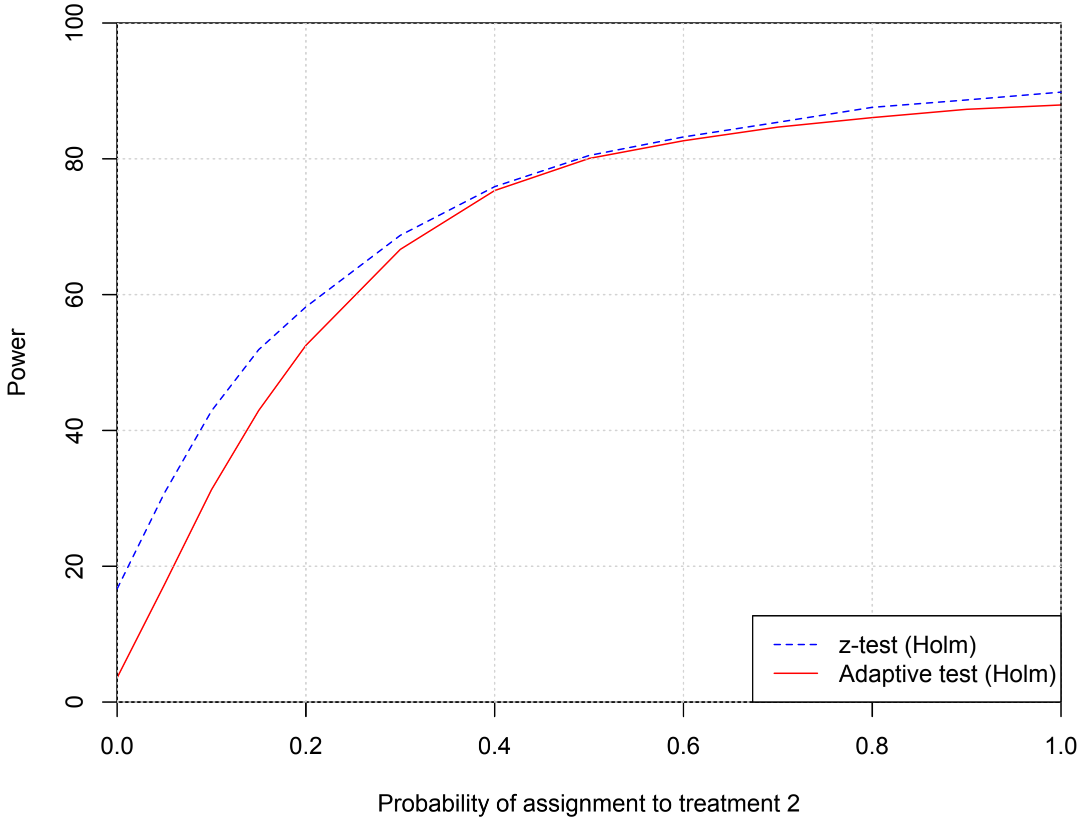

Figure 2: *Power of the Holm adaptive test and Holm z-test as a function of the probability of assignment to treatment 2. We use the block randomized trial setup of Section 4.3 in the paper, with $t = 2$ treatments and $\delta_1 = 0$, $\delta_2 = 0.5$.*

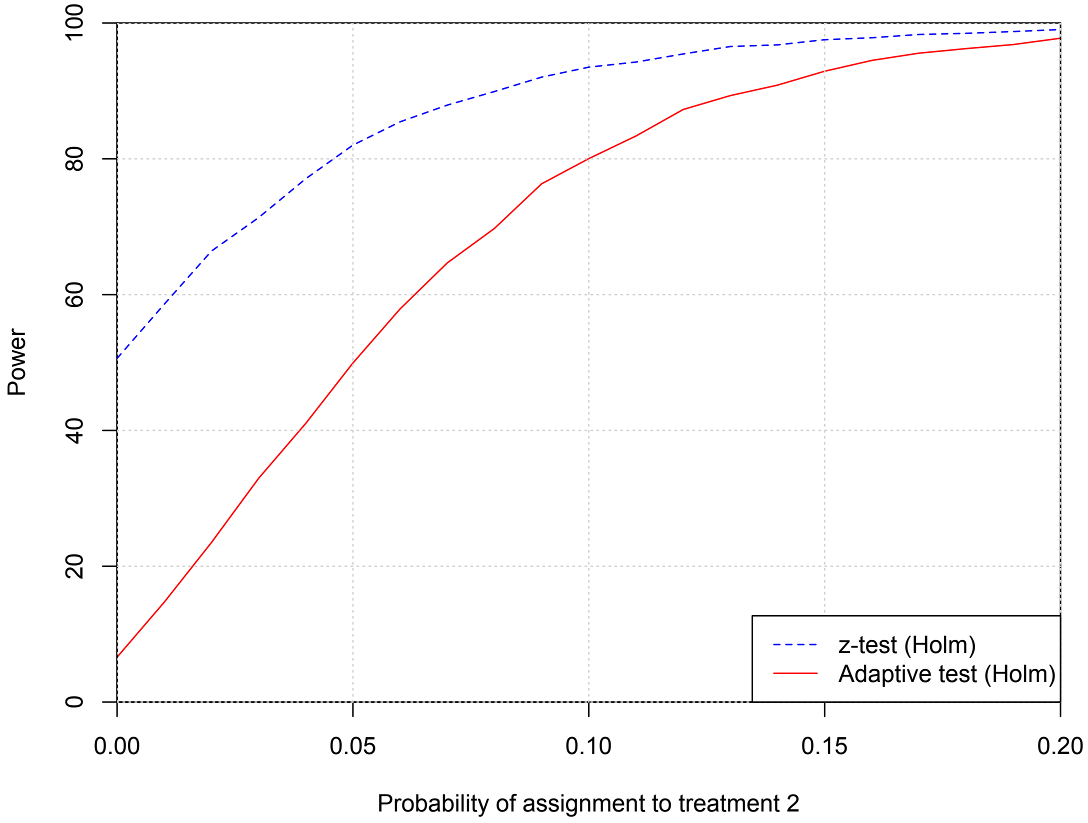

Figure 3: *Power of the Holm adaptive test and Holm z-test as a function of the probability of assignment to treatment 2. We use the block randomized trial setup of Section 4.3 in the paper, with* $t = 2$ *treatments and* $\delta_1 = 0$, $\delta_2 = 1$.

F.5 Power comparison with equal randomization

In order to see how the adaptive randomization procedures affect power, we provide a comparison with using equal randomization. More precisely, after the burn-in period patients are assigned to each of the experimental treatments (as well as tp the control arm, if there is adaptive allocation to the control) with equal probability. Since the type I error inflator would never be used in practice, we only compare equal randomization with BAR in our comments below.

F.5.1 Fully sequential randomization (with a fixed control allocation)

Table 8 gives the results for the fully sequential setting. Compared with using BAR (see Table 3 in the paper), BAR has a higher disjunctive power for all testing procedures and scenarios (except for scenario 8 for the Holm and Bonferroni z-tests). In particular, there is a substantial increase in the power for the closed testing procedures in scenarios where there is at least one true null hypothesis.

Table 8: *Familywise error rate and disjunctive power for equal randomization in the fully sequential setting. There were* 10^5 *simulated trials for each set of parameter values.*

	Adaptive closed test		Adaptive test (Holm)		Closed z-test		z-test (Holm)		z-test (Bonferroni)	
Parameter values	Error	Power	Error	Power	Error	Power	Error	Power	Error	Power
1. $\delta_1 = \delta_2 = 0$	4.3	-	4.3	-	4.6	-	4.5	-	4.5	-
2. $\delta_1 = 0$, $\delta_2 = 0.5$	4.7	29.0	4.3	46.3	4.9	29.4	4.6	48.9	2.5	48.8
3. $\delta_1 = \delta_2 = 0.5$	-	69.1	-	63.4	-	70.6	-	65.5	-	65.5
4. $\delta_1 = \delta_2 = \delta_3 = 0$	3.7	-	4.1	-	4.0	-	4.3	-	4.3	
5. $\delta_1 = \delta_2 = 0$, $\delta_3 = 1$	4.3	35.7	4.3	81.4	4.7	36.3	4.6	85.9	3.1	85.9
6. $\delta_1 = 0$, $\delta_2 = \delta_3 = 1$	4.7	67.2	4.7	92.7	5.0	67.5	5.0	94.8	1.7	94.8
7. $\delta_1 = 0$, $\delta_2 = 0.5$, $\delta_3 = 1$	4.6	54.2	4.0	83.1	4.9	55.3	4.4	86.9	1.7	86.9
8. $\delta_1 = \delta_2 = \delta_3 = 0.5$	-	55.1	-	49.0	-	57.3	-	52.8	-	52.8

F.5.2 Block randomization with a fixed control allocation

Table 9 gives the results for block randomization with a fixed control allocation. Compared with using BAR (see Table 5 in the paper), BAR has a higher disjunctive power for all testing procedures and in all scenarios. This increase in power is particularly noticeable for the closed testing procedures in scenarios where there is at least one true null hypothesis.

Table 9: *Familywise error rate and disjunctive power for equal randomization, for block randomization with a fixed control allocation. There were* 10^5 *simulated trials for each set of parameter values.*

	Adaptive closed test		Adaptive test (Holm)		Closed z-test		z-test (Holm)		z-test (Bonferroni)	
Parameter values	Error	Power	Error	Power	Error	Power	Error	Power	Error	Power
1. $\delta_1 = \delta_2 = 0$	4.6	-	4.5	-	4.7	-	4.6	-	4.6	-
2. $\delta_1 = 0$, $\delta_2 = 0.5$	4.8	50.0	4.7	80.0	4.9	50.1	4.8	80.4	2.4	80.3
3. $\delta_1 = \delta_2 = 0.5$	-	94.3	-	91.8	-	94.4	-	92.0	-	92.0
4. $\delta_1 = \delta_2 = \delta_3 = 0$	3.7	-	4.5	-	3.7	-	4.5	-	4.5	
5. $\delta_1 = \delta_2 = 0$, $\delta_3 = 0.5$	4.5	27.5	4.5	66.5	4.5	27.6	4.6	67.1	3.2	67.0
6. $\delta_1 = 0$, $\delta_2 = \delta_3 = 0.5$	5.0	56.0	4.7	83.6	4.9	56.0	4.6	83.8	1.7	83.8
7. $\delta_1 = 0$, $\delta_2 = 0.25$, $\delta_3 = 0.5$	4.6	42.9	3.7	69.9	4.7	43.0	3.7	70.3	1.6	70.3
8. $\delta_1 = \delta_2 = \delta_3 = 0.5$	-	93.2	-	90.0	-	93.2	-	90.2	-	90.2

F.5.3 Block randomization with an adaptive control allocation

Table 10 gives the results for block randomization with an adaptive control allocation. Compared with using BAR (see Table 5 in Web Appendix F.3), BAR again has a higher disjunctive power for all testing procedures and in all scenarios. This increase in power is most noticeable for the closed testing procedures in scenarios where there is at least one true null hypothesis.

Table 10: *Familywise error rate and disjunctive power for equal randomization, for block randomization with an adaptive control allocation. There were* 10^5 *simulated trials for each set of parameter values.*

	Adaptive closed test		Adaptive test (Holm)		Closed z-test		z-test (Holm)		z-test (Bonferroni)	
Parameter values	Error	Power	Error	Power	Error	Power	Error	Power	Error	Power
1. $\delta_1 = \delta_2 = 0$	4.6	-	4.5	-	4.6	-	4.5	-	4.5	-
2. $\delta_1 = 0$, $\delta_2 = 1$	5.0	44.1	4.9	73.4	5.0	44.5	4.9	74.2	2.9	74.2
3. $\delta_1 = \delta_2 = 0.5$	-	90.3	-	86.9	-	90.7	-	87.5	-	87.5
4. $\delta_1 = \delta_2 = \delta_3 = 0$	3.9	-	4.3	-	3.9	-	4.3	-	4.3	-
5. $\delta_1 = \delta_2 = 0$, $\delta_3 = 0.5$	4.5	22.2	4.4	55.5	4.6	22.6	4.4	56.7	3.0	56.7
6. $\delta_1 = 0$, $\delta_2 = \delta_3 = 0.5$	4.9	45.5	4.6	71.9	5.0	45.9	4.6	72.8	1.6	72.8
7. $\delta_1 = 0$, $\delta_2 = 0.25$, $\delta_3 = 0.5$	4.6	34.3	3.8	57.7	4.6	34.7	3.8	58.8	1.7	58.8
8. $\delta_1 = \delta_2 = \delta_3 = 0.5$	-	84.2	-	79.6	-	84.8	-	80.5	-	80.5

F.6 Using the pooled sample variance

In this section, we assume that the common variance σ^2 is unknown and will be estimated at the end of the trial using the pooled sample variance $\hat{\sigma}^2$ of the experimental treatments and control. Given that there are t experimental treatments, the formula for the pooled sample variance is

$$\hat{\sigma}^2 = \frac{\sum_{i=0}^{t}(n_i - 1)s_i^2}{\sum_{i=0}^{t}(n_i - 1)} = \frac{\sum_{i=0}^{t}(n_i - 1)s_i^2}{n - t - 1}$$

where s_i^2 is the sample variance for treatment i ($i = 0, 1, \ldots, t$), as defined below. For an adaptive control allocation,

$$s_i^2 = \frac{1}{n_i - 1}\sum_{k=1}^{n}\left(\mathbb{1}_{\{a_k = i\}}\left(X_k - \bar{X}_i\right)^2\right) \qquad \text{for } i = 0, 1, \ldots, t$$

where

$$\bar{X}_i = \frac{1}{n_i}\sum_{k=1}^{n}\mathbb{1}_{\{a_k = i\}}X_k \qquad \text{for } i = 0, 1, \ldots, t$$

For a fixed control allocation, the formulae for s_i^2 and $\bar{X}_i$ are the same for $i = 1, \ldots, t$, but now

$$s_0^2 = \frac{1}{n_0 - 1}\sum_{j=1}^{n_0}\left(X_{0j} - \bar{X}_0\right)^2 \quad \text{and} \quad \bar{X}_0 = \frac{1}{n_0}\sum_{j=1}^{n_0}X_{0j}$$

Estimating the pooled variance from the data in this way means using a t-test (rather than a z-test) for H_I, i.e. we reject H_I if the test statistic $T_I/\hat{\sigma}$ is greater than $t_{d,\alpha}(1/n_I + 1/n_0)^{1/2}$, where $t_{d,\alpha}$ is that $(1-\alpha)$ quantile of a t-distribution with $d = n - t - 1$ degrees of freedom. The p-values derived from this t-test are then $p_I = 1 - F_{t_d}((1/n_i + 1/n_0)^{-1/2}\, T_i/\hat{\sigma})$, where F_{t_d} is the cdf of a t-distribution with d degrees of freedom. As a natural comparison with our adaptive test procedure, we make the same adjustment: given the adaptive test statistic $\tilde{T}_I$, to test hypothesis H_I we compare $\tilde{T}_I/\hat{\sigma}$ with the critical value $t_{d,\alpha}(1/n'_I + 1/n'_0)^{1/2}$. The adjusted p-values are then $\tilde{p}_I = 1 - F_{t_d}((1/n'_i + 1/n'_0)^{-1/2}\, \tilde{T}_i/\hat{\sigma})$.

We rerun all the simulation studies in Section 4 of the paper and Web Appendix F.3, with exactly the same setup except for the above changes to the test procedures. Due to the extra variability induced by using the pooled sample variance, we simulate 10^6 trials for each set of parameter values. As the z-tests are now t-tests, we make the corresponding change to the names of the procedures.

F.6.1 Fully sequential randomization (with a fixed control allocation)

Table 11 gives the results for the type I error inflator, and Table 12 the results for BAR. Compared with assuming a common known variance of $\sigma^2 = 1$ (see Tables 2 and 3 in the paper), for almost all scenarios and testing procedures there is a small decrease in power (as would be expected). For the type I error inflator, the same testing procedures as before inflate the FWER, except that this time the Bonferroni-corrected t-test now has a FWER slightly above 5% in scenario 2. Both adaptive tests still maintain strong FWER control in all scenarios. For BAR, there is a slight decrease in the FWER for all scenarios, with all the testing strategies controlling the FWER.

Table 11: *Familywise error rate and disjunctive power for the type I error inflator in the fully sequential setting. There were 10^6 simulated trials for each set of parameter values.*

	Adaptive closed test		Adaptive test (Holm)		Closed t-test		t-test (Holm)		t-test (Bonferroni)	
Parameter values	Error	Power	Error	Power	Error	Power	Error	Power	Error	Power
1. $\delta_1 = \delta_2 = 0$	3.3	-	4.6	-	4.7	-	**7.0**	-	**7.0**	-
2. $\delta_1 = 0$, $\delta_2 = 1$	4.8	21.7	3.7	27.2	**10.2**	26.4	**9.8**	63.0	**5.1**	62.9
3. $\delta_1 = \delta_2 = 0.5$	-	61.7	-	51.4	-	69.2	-	60.5	-	60.5
4. $\delta_1 = \delta_2 = \delta_3 = 0$	2.9	-	3.9	-	4.1	-	**5.8**	-	**5.8**	
5. $\delta_1 = \delta_2 = 0$, $\delta_3 = 1$	3.4	13.0	4.3	24.4	**5.2**	17.1	**6.6**	53.5	4.6	53.4
6. $\delta_1 = 0$, $\delta_2 = \delta_3 = 1$	4.9	22.3	3.4	28.1	**9.9**	27.1	**9.2**	71.4	3.3	71.4
7. $\delta_1 = 0$, $\delta_2 = 0.5$, $\delta_3 = 1$	4.3	19.3	2.8	24.9	**9.2**	24.1	**7.4**	57.5	3.3	57.5
8. $\delta_1 = \delta_2 = \delta_3 = 0.5$	-	50.8	-	41.4	-	56.9	-	48.2	-	48.2

Table 12: *Familywise error rate and disjunctive power for BAR in the fully sequential setting. There were 10^6 simulated trials for each set of parameter values.*

	Adaptive closed test		Adaptive test (Holm)		Closed t-test		t-test (Holm)		t-test (Bonferroni)	
Parameter values	Error	Power	Error	Power	Error	Power	Error	Power	Error	Power
1. $\delta_1 = \delta_2 = 0$	4.7	-	4.4	-	4.7	-	4.0	-	4.0	-
2. $\delta_1 = 0$, $\delta_2 = 0.5$	4.5	46.0	4.2	51.5	3.8	46.1	3.5	52.5	1.9	52.4
3. $\delta_1 = \delta_2 = 0.5$	-	70.1	-	65.4	-	70.4	-	64.6	-	64.6
4. $\delta_1 = \delta_2 = \delta_3 = 0$	3.9	-	4.2	-	4.0	-	3.8	-	3.8	
5. $\delta_1 = \delta_2 = 0$, $\delta_3 = 1$	4.5	59.1	4.5	88.1	4.4	59.3	3.9	89.6	2.6	89.5
6. $\delta_1 = 0$, $\delta_2 = \delta_3 = 1$	4.9	89.4	4.8	94.8	4.0	89.4	4.0	95.4	1.3	95.4
7. $\delta_1 = 0$, $\delta_2 = 0.5$, $\delta_3 = 1$	4.5	74.5	4.0	87.9	3.9	75.1	3.4	89.0	1.3	89.0
8. $\delta_1 = \delta_2 = \delta_3 = 0.5$	-	56.3	-	51.5	-	57.1	-	51.1	-	51.1

F.6.2 Block randomization with a fixed control allocation

Table 13 gives the results for the type I error inflator, and Table 14 the results for BAR. Compared with assuming a common known variance of $\sigma^2 = 1$ (see Tables 4 and 5 in the paper), in all scenarios the power decreases slightly (as would be expected). For the type I error inflator, the same testing procedures as before lead to an inflation of the FWER, while both the adaptive closed test and the Holm adaptive test maintain FWER control. For BAR, as before all the testing procedures control the FWER (which also decreases slightly in all scenarios).

Table 13: *Familywise error rate and disjunctive power for the type I error inflator, for block randomization with a fixed control allocation. There were* 10^6 *simulated trials for each set of parameter values.*

	Adaptive closed test		Adaptive test (Holm)		Closed t-test		t-test (Holm)		t-test (Bonferroni)	
Parameter values	Error	Power	Error	Power	Error	Power	Error	Power	Error	Power
1. $\delta_1 = \delta_2 = 0$	3.8	-	4.7	-	4.7	-	**6.7**	-	**6.7**	-
2. $\delta_1 = 0$, $\delta_2 = 1$	4.8	21.9	3.6	26.9	**8.3**	25.6	**7.8**	60.7	4.3	60.5
3. $\delta_1 = \delta_2 = 0.5$	-	92.5	-	87.4	-	94.5	-	91.4	-	91.4
4. $\delta_1 = \delta_2 = \delta_3 = 0$	3.2	-	4.0	-	4.1	-	**6.1**	-	**6.1**	
5. $\delta_1 = \delta_2 = 0$, $\delta_3 = 1$	3.7	14.2	4.3	23.2	4.8	18.2	**6.2**	61.0	4.5	60.8
6. $\delta_1 = 0$, $\delta_2 = \delta_3 = 1$	4.9	20.0	3.2	25.9	**8.2**	23.0	**7.4**	79.4	2.9	79.3
7. $\delta_1 = 0$, $\delta_2 = 0.5$, $\delta_3 = 1$	4.7	17.7	2.9	23.7	**8.0**	21.2	**6.7**	65.9	2.9	65.9
8. $\delta_1 = \delta_2 = \delta_3 = 0.5$	-	91.1	-	82.7	-	93.8	-	89.1	-	89.1

Table 14: *Familywise error rate and disjunctive power for BAR, for block randomization with a fixed control allocation. There were* 10^6 *simulated trials for each set of parameter values. There were* 10^6 *simulated trials for each set of parameter values.*

	Adaptive closed test		Adaptive test (Holm)		Closed t-test		t-test (Holm)		t-test (Bonferroni)	
Parameter values	Error	Power	Error	Power	Error	Power	Error	Power	Error	Power
1. $\delta_1 = \delta_2 = 0$	4.6	-	4.4	-	4.6	-	4.4	-	4.4	-
2. $\delta_1 = 0$, $\delta_2 = 0.5$	4.9	60.9	4.9	82.3	4.9	60.9	4.8	82.6	2.4	82.6
3. $\delta_1 = \delta_2 = 0.5$	-	94.4	-	91.9	-	94.4	-	91.9	-	91.9
4. $\delta_1 = \delta_2 = \delta_3 = 0$	3.7	-	4.5	-	3.7	-	4.2	-	4.2	
5. $\delta_1 = \delta_2 = 0$, $\delta_3 = 0.5$	4.5	35.8	4.5	71.2	4.4	35.8	4.4	71.2	3.0	71.2
6. $\delta_1 = 0$, $\delta_2 = \delta_3 = 0.5$	5.0	66.9	4.6	85.1	4.7	66.5	4.4	84.9	1.6	84.9
7. $\delta_1 = 0$, $\delta_2 = 0.25$, $\delta_3 = 0.5$	4.5	50.6	3.6	72.3	4.3	50.4	3.5	72.0	1.6	72.0
8. $\delta_1 = \delta_2 = \delta_3 = 0.5$	-	93.1	-	90.1	-	93.1	-	89.9	-	89.9

F.6.3 Block randomisation with an adaptive control allocation

Table 15 gives the results for the type I error inflator, and Table 16 the results for BAR. Compared with assuming a common known variance of $\sigma^2 = 1$ (see Web Appendix F.3), in all scenarios the power decreases slightly. For the type I error inflator, the same testing procedures as before lead to an inflation of the FWER, while both the adaptive closed test and the Holm adaptive test maintain FWER control. For BAR, again all the testing procedures control the FWER (which also slightly decreases in all scenarios).

Table 15: *Familywise error rate and disjunctive power for the type I error inflator, for block randomization with an adaptive control allocation. There were* 10^6 *simulated trials for each set of parameter values.*

	Adaptive closed test		Adaptive test (Holm)		Closed t-test		t-test (Holm)		t-test (Bonferroni)	
Parameter values	Error	Power	Error	Power	Error	Power	Error	Power	Error	Power
1. $\delta_1 = \delta_2 = 0$	3.8	-	4.8	-	4.5	-	**6.7**	-	**6.7**	-
2. $\delta_1 = 0$, $\delta_2 = 1$	4.9	19.1	3.7	24.9	**8.2**	25.6	**7.8**	66.9	4.3	66.8
3. $\delta_1 = \delta_2 = 0.5$	-	89.9	-	84.0	-	93.2	-	89.5	-	89.5
4. $\delta_1 = \delta_2 = \delta_3 = 0$	3.2	-	4.0	-	3.8	-	**6.3**	-	**6.3**	-
5. $\delta_1 = \delta_2 = 0$, $\delta_3 = 1$	3.7	13.9	4.3	21.9	4.8	19.9	**6.4**	61.4	4.7	61.3
6. $\delta_1 = 0$, $\delta_2 = \delta_3 = 1$	4.9	19.1	3.4	24.5	**8.3**	26.3	**7.5**	80.1	3.0	80.0
7. $\delta_1 = 0$, $\delta_2 = 0.5$, $\delta_3 = 1$	4.6	17.0	3.0	22.3	**8.1**	23.8	**6.6**	66.4	3.0	66.3
8. $\delta_1 = \delta_2 = \delta_3 = 0.5$	-	87.3	-	77.8	-	91.7	-	86.4	-	86.4

Table 16: *Familywise error rate and disjunctive power for BAR, for block randomization with an adaptive control allocation. There were* 10^6 *simulated trials for each set of parameter values.*

	Adaptive closed test		Adaptive test (Holm)		Closed t-test		t-test (Holm)		t-test (Bonferroni)	
Parameter values	Error	Power	Error	Power	Error	Power	Error	Power	Error	Power
1. $\delta_1 = \delta_2 = 0$	4.6	-	4.5	-	4.6	-	4.5	-	4.5	-
2. $\delta_1 = 0$, $\delta_2 = 0.5$	5.0	54.1	4.9	76.0	4.8	55.7	4.7	77.6	2.4	77.6
3. $\delta_1 = \delta_2 = 0.5$	-	90.3	-	86.9	-	91.3	-	87.9	-	87.9
4. $\delta_1 = \delta_2 = \delta_3 = 0$	3.9	-	4.3	-	3.9	-	4.2	-	4.2	-
5. $\delta_1 = \delta_2 = 0$, $\delta_3 = 0.5$	4.6	28.9	4.4	60.7	4.5	29.8	4.3	62.3	3.0	62.2
6. $\delta_1 = 0$, $\delta_2 = \delta_3 = 0.5$	4.9	56.2	4.5	75.3	4.8	57.1	4.4	76.5	1.6	76.5
7. $\delta_1 = 0$, $\delta_2 = 0.25$, $\delta_3 = 0.5$	4.4	41.4	3.6	61.8	4.4	42.5	3.5	63.0	1.6	63.0
8. $\delta_1 = \delta_2 = \delta_3 = 0.5$	-	85.7	-	81.3	-	86.7	-	82.4	-	82.4

F.7 Using the sample variance when the variances are unequal

We now consider the more general setting where the variances of the experimental treatments and the control are not equal, and are estimated from the data. More precisely, we assume the efficacy outcomes are distributed as follows:

$$X_{0j} \sim N(\mu, \sigma_0^2), \quad X_k\,|_{a_k=i} \sim N(\mu + \delta_i, \sigma_i^2)$$

Hence the distribution of the test statistic T_I under the null is

$$T_I \sim N\left(0\,,\, \frac{\sigma_0^2}{n_0} + \frac{\sum_{i\in I} n_i \sigma_i^2}{n_I^2}\right)$$

The Welch t-statistic used to test H_I is $T_I/\sqrt{\chi_I}$, where

$$\chi_I = \frac{s_0^2}{n_0} + \frac{\sum_{i\in I} n_i s_i^2}{n_I^2}$$

We reject H_I if $T_I/\sqrt{\chi_I}$ is greater than $t_{d,\alpha}$, where $t_{d,\alpha}$ is the $(1-\alpha)$ quantile of a t-distribution with d degrees of freedom, where (using the Welch–Satterthwaite equation)

$$d = \frac{\chi_I^2}{\dfrac{(s_0^2/n_0)^2}{n_0 - 1} + \displaystyle\sum_{i\in I} \frac{(n_i s_i^2/n_I^2)^2}{n_i - 1}}$$

The p-values derived from this t-test are then $p_I = 1 - F_{t_d}(T_i/\sqrt{\chi_I})$. Note that Welch's test is only approximate and not an exact test.

In this setting, making a comparison with our adaptive test procedure is not straightforward, as our approach requires equal variances to work. Hence we instead resort to using a large-sample approximation that $s_i \approx \sigma_i$ (for $i = 0, 1, \ldots, t$) and use the re-scaled efficacy outcomes

$$\tilde{X}_{0j} = \frac{X_{0j}}{s_0}, \quad \tilde{X}_k\,|_{a_k=i} = \frac{X_k}{s_i}$$

With these transformed outcomes, to test hypothesis H_I we compare $\tilde{T}_I$ with the critical value $t_{d,\alpha}(1/n'_I + 1/n'_0)^{1/2}$. The adjusted p-values are $\tilde{p}_I = 1 - F_{t_d}((1/n'_I + 1/n'_0)^{-1/2}\,\tilde{T}_I)$.

We rerun all the simulation studies in Section 4 of the paper, with exactly the same setup except for the above changes to the test procedures. Due to the extra variability induced by using the sample variance, we simulate 10^6 trials for each set of parameter values. As the z-tests are now t-tests, we make the corresponding change to the names of the relevant procedures.

F.7.1 Fully sequential randomization (with a fixed control allocation)

Table 17 gives the results for the type I error inflator, and Table 18 the results for BAR. Compared with assuming a common known variance of $\sigma^2 = 1$ (see Tables 2 and 3 in the paper), the power is reduced in all scenarios (as would be expected). In particular, there is a substantial drop in power for the Holm and Bonferroni t-tests in scenarios 2, 6 and 7 for the type I eror inflator. The same testing procedures as before lead to an inflation of the FWER for the type I error inflator, while both adaptive tests maintain FWER control. For BAR, all the testing procedures control the FWER for all scenarios.

Table 17: *Familywise error rate and disjunctive power for the type I error inflator in the fully sequential setting. There were* 10^6 *simulated trials for each set of parameter values.*

	Adaptive closed test		Adaptive test (Holm)		Closed t-test		t-test (Holm)		t-test (Bonferroni)	
Parameter values	Error	Power	Error	Power	Error	Power	Error	Power	Error	Power
1. $\delta_1 = \delta_2 = 0$	3.3	-	3.4	-	4.5	-	**7.3**	-	**7.3**	-
2. $\delta_1 = 0$, $\delta_2 = 1$	4.6	21.5	3.4	26.1	**9.3**	25.9	**8.7**	53.2	4.9	53.0
3. $\delta_1 = \delta_2 = 0.5$	-	60.9	-	51.3	-	68.6	-	60.0	-	60.0
4. $\delta_1 = \delta_2 = \delta_3 = 0$	3.0	-	3.2	-	4.1	-	**6.6**	-	**6.6**	
5. $\delta_1 = \delta_2 = 0$, $\delta_3 = 1$	3.8	12.4	3.3	23.7	4.9	16.3	**6.5**	44.7	5.0	44.4
6. $\delta_1 = 0$, $\delta_2 = \delta_3 = 1$	5.0	22.7	3.3	29.8	**9.2**	26.8	**8.0**	62.3	3.3	62.2
7. $\delta_1 = 0$, $\delta_2 = 0.5$, $\delta_3 = 1$	4.5	19.0	2.7	25.3	**8.3**	23.5	**6.5**	49.7	3.3	49.6
8. $\delta_1 = \delta_2 = \delta_3 = 0.5$	-	49.7	-	42.5	-	56.0	-	47.6	-	47.6

Table 18: *Familywise error rate and disjunctive power for BAR in the fully sequential setting. There were* 10^6 *simulated trials for each set of parameter values.*

	Adaptive closed test		Adaptive test (Holm)		Closed t-test		t-test (Holm)		t-test (Bonferroni)	
Parameter values	Error	Power	Error	Power	Error	Power	Error	Power	Error	Power
1. $\delta_1 = \delta_2 = 0$	4.7	-	4.6	-	4.7	-	4.0	-	4.0	-
2. $\delta_1 = 0$, $\delta_2 = 0.5$	4.6	45.4	4.3	51.8	3.9	45.8	3.6	52.0	2.0	51.9
3. $\delta_1 = \delta_2 = 0.5$	-	69.0	-	66.3	-	70.1	-	64.3	-	64.3
4. $\delta_1 = \delta_2 = \delta_3 = 0$	4.0	-	4.4	-	4.0	-	3.7	-	3.7	
5. $\delta_1 = \delta_2 = 0$, $\delta_3 = 1$	5.0	57.0	4.8	85.2	4.4	58.3	4.0	87.5	2.7	87.5
6. $\delta_1 = 0$, $\delta_2 = \delta_3 = 1$	4.8	88.0	4.7	94.4	4.1	88.9	4.0	94.8	1.3	94.8
7. $\delta_1 = 0$, $\delta_2 = 0.5$, $\delta_3 = 1$	4.6	71.6	4.0	86.4	3.9	74.1	3.4	87.8	1.3	87.8
8. $\delta_1 = \delta_2 = \delta_3 = 0.5$	-	54.3	-	53.1	-	56.3	-	49.9	-	49.9

F.7.2 Block randomization with a fixed control allocation

Table 19 gives the results for the type I error inflator, and Table 20 the results for BAR. Compared with assuming a common known variance of $\sigma^2 = 1$ (see Tables 4 and 5 in the paper), there is a decrease in power in all scenarios (as would be expected). In particular, there is a substantial decrease in the power of the Holm and Bonferroni t-tests for the type I error inflator except when all hypotheses are non-null (scenarios 3 and 8). The same testing procedures as before lead to an inflation of the FWER for the type I error inflator, while both adaptive tests maintain FWER control. For BAR, all the testing procedures control the FWER for all scenarios.

Table 19: *Familywise error rate and disjunctive power for the type I error inflator, for block randomization with a fixed control allocation. There were 10^6 simulated trials for each set of parameter values.*

	Adaptive closed test		Adaptive test (Holm)		Closed t-test		t-test (Holm)		t-test (Bonferroni)	
Parameter values	Error	Power	Error	Power	Error	Power	Error	Power	Error	Power
1. $\delta_1 = \delta_2 = 0$	3.8	-	3.3	-	4.6	-	**6.7**	-	**6.7**	-
2. $\delta_1 = 0$, $\delta_2 = 1$	4.3	21.8	3.0	24.9	**7.6**	24.9	**6.8**	48.7	4.1	48.5
3. $\delta_1 = \delta_2 = 0.5$	-	91.9	-	87.0	-	94.3	-	91.1	-	91.1
4. $\delta_1 = \delta_2 = \delta_3 = 0$	3.2	-	2.8	-	4.0	-	**6.6**	-	**6.6**	
5. $\delta_1 = \delta_2 = 0$, $\delta_3 = 1$	3.8	14.1	2.9	21.5	4.7	17.7	**6.1**	47.7	4.8	47.5
6. $\delta_1 = 0$, $\delta_2 = \delta_3 = 1$	4.6	20.3	2.8	25.0	**7.4**	22.6	**6.2**	64.5	2.8	64.5
7. $\delta_1 = 0$, $\delta_2 = 0.5$, $\delta_3 = 1$	4.4	17.7	2.6	22.4	**7.4**	20.7	**5.8**	53.3	2.9	53.2
8. $\delta_1 = \delta_2 = \delta_3 = 0.5$	-	90.6	-	82.5	-	93.7	-	88.7	-	88.7

Table 20: *Familywise error rate and disjunctive power for BAR, for block randomization with a fixed control allocation. There were 10^6 simulated trials for each set of parameter values. There were 10^6 simulated trials for each set of parameter values.*

	Adaptive closed test		Adaptive test (Holm)		Closed t-test		t-test (Holm)		t-test (Bonferroni)	
Parameter values	Error	Power	Error	Power	Error	Power	Error	Power	Error	Power
1. $\delta_1 = \delta_2 = 0$	4.6	-	4.6	-	4.6	-	4.4	-	4.4	-
2. $\delta_1 = 0$, $\delta_2 = 0.5$	5.0	60.7	5.0	60.7	4.8	60.7	4.8	82.2	2.4	82.2
3. $\delta_1 = \delta_2 = 0.5$	-	93.9	-	91.8	-	94.3	-	91.8	-	91.8
4. $\delta_1 = \delta_2 = \delta_3 = 0$	3.8	-	4.6	-	3.7	-	4.2	-	4.2	
5. $\delta_1 = \delta_2 = 0$, $\delta_3 = 0.5$	4.7	35.1	4.7	69.9	4.4	35.5	4.3	69.8	3.0	69.8
6. $\delta_1 = 0$, $\delta_2 = \delta_3 = 0.5$	5.0	66.9	4.7	85.1	4.7	66.3	4.4	84.5	1.6	84.5
7. $\delta_1 = 0$, $\delta_2 = 0.25$, $\delta_3 = 0.5$	4.6	50.0	3.7	72.0	4.3	50.2	3.5	71.4	1.6	71.4
8. $\delta_1 = \delta_2 = \delta_3 = 0.5$	-	92.7	-	90.3	-	93.0	-	89.6	-	89.6

F.7.3 Block randomization with an adaptive control allocation

Table 21 gives the results for the type I error inflator, and Table 22 the results for BAR. Compared with assuming a common known variance of $\sigma^2 = 1$ (see Web Appendix F.3), there is a decrease in power in all scenarios (as would be expected). In particular, there is a substantial decrease in the power of the Holm and Bonferroni t-tests for the type I error inflator except when all hypotheses are non-null (scenarios 3 and 8). The same testing procedures as before inflate the FWER for the type I error inflator, while both adaptive tests maintain FWER control. This time, for BAR the adaptive closed test leads to a slight inflation of the FWER in scenarios 2 and 6. However, the Holm adaptive test still maintains strong FWER control, as do the other testing procedures.

Table 21: *Familywise error rate and disjunctive power for the type I error inflator, for block randomization with an adaptive control allocation. There were 10^6 simulated trials for each set of parameter values.*

	Adaptive closed test		Adaptive test (Holm)		Closed t-test		t-test (Holm)		t-test (Bonferroni)	
Parameter values	Error	Power	Error	Power	Error	Power	Error	Power	Error	Power
1. $\delta_1 = \delta_2 = 0$	3.9	-	3.4	-	4.5	-	**6.8**	-	**6.8**	-
2. $\delta_1 = 0$, $\delta_2 = 1$	4.6	19.5	3.3	22.9	**7.5**	24.8	**6.8**	54.3	4.1	54.1
3. $\delta_1 = \delta_2 = 0.5$	-	89.8	-	83.7	-	92.9	-	89.1	-	89.1
4. $\delta_1 = \delta_2 = \delta_3 = 0$	3.3	-	2.7	-	3.8	-	**6.8**	-	**6.8**	-
5. $\delta_1 = \delta_2 = 0$, $\delta_3 = 1$	3.8	14.3	2.9	20.3	4.7	19.4	**6.3**	47.8	4.9	47.6
6. $\delta_1 = 0$, $\delta_2 = \delta_3 = 1$	4.6	20.1	3.0	23.3	**7.7**	25.9	**6.5**	64.6	3.0	64.5
7. $\delta_1 = 0$, $\delta_2 = 0.5$, $\delta_3 = 1$	4.4	17.6	2.6	20.8	**7.4**	23.2	**5.8**	53.2	3.0	53.1
8. $\delta_1 = \delta_2 = \delta_3 = 0.5$	-	87.4	-	77.8	-	91.4	-	85.8	-	85.8

Table 22: *Familywise error rate and disjunctive power for BAR, for block randomization with an adaptive control allocation. There were 10^6 simulated trials for each set of parameter values.*

	Adaptive closed test		Adaptive test (Holm)		Closed t-test		t-test (Holm)		t-test (Bonferroni)	
Parameter values	Error	Power	Error	Power	Error	Power	Error	Power	Error	Power
1. $\delta_1 = \delta_2 = 0$	4.8	-	4.7	-	4.6	-	4.4	-	4.4	-
2. $\delta_1 = 0$, $\delta_2 = 0.5$	**5.1**	54.5	5.0	75.9	4.8	55.6	4.7	77.5	2.4	77.4
3. $\delta_1 = \delta_2 = 0.5$	-	90.3	-	87.1	-	91.2	-	87.8	-	87.8
4. $\delta_1 = \delta_2 = \delta_3 = 0$	4.1	-	4.5	-	3.9	-	4.2	-	4.2	-
5. $\delta_1 = \delta_2 = 0$, $\delta_3 = 0.5$	4.8	29.3	4.6	59.7	4.5	29.7	4.3	60.8	3.0	60.7
6. $\delta_1 = 0$, $\delta_2 = \delta_3 = 0.5$	**5.1**	57.3	4.7	75.7	4.8	57.0	4.4	76.2	1.6	76.1
7. $\delta_1 = 0$, $\delta_2 = 0.25$, $\delta_3 = 0.5$	4.6	42.0	3.8	61.6	4.4	42.2	3.5	62.1	1.6	62.1
8. $\delta_1 = \delta_2 = \delta_3 = 0.5$	-	85.9	-	82.0	-	86.4	-	82.0	-	82.0

References

Athreya, K. B. and Karlin, S. (1968). Embedding of urn schemes into continuous time markov branching processes and related limit theorems. *The Annals of Mathematical Statistics* **39,** 1801–1817.

Bello, G. A. and Sabo, R. T. (2016). Outcome-adaptive allocation with natural lead-in for three-group trials with binary outcomes. *Journal of Statistical Computation and Simulation* **86,** 2441–2449.

Bergemann, D. and Vlimki, J. (2006). Bandit problems. Technical report, Cowles Foundation, http://ssrn.com/abstract=877173 [accessed 8 Jan 2019].

Berry, D. A. (2011). Adaptive clinical trials: the promise and the caution. *Journal of Clinical Oncology* **29,** 606–609.

Berry, D. A. (2015). Commentary on Hey and Kimmelman. *Clinical Trials* **12,** 107–109.

Biswas, A. and Bhattacharya, R. (2016). Response-adaptive designs for continuous treatment responses in phase III clinical trials: A review. *Statistical Methods in Medical Research* **25,** 81–100.

Biswas, A., Bhattacharya, R., and Zhang, L. (2007). Optimal response-adaptive designs for continuous responses in phase III trials. *Biometrical Journal* **49,** 928–940.

Du, Y., Wang, X., and Lee, J. J. (2015). Simulation study for evaluating the performance of response-adaptive randomization. *Contemporary Clinical Trials* **40,** 15–25.

Eisele, J. R. (1994). The doubly adaptive biased coin design for sequential clinical trials. *Journal of Statistical Planning and Inference* **38,** 249–261.

Hey, S. P. and Kimmelman, J. (2015). Are outcome-adaptive allocation trials ethical? *Clinical Trials* **12,** 102–106.

Hu, F. and Rosenberger, W. F. (2006). *The theory of response-adaptive randomization in clinical trials.* John Wiley & Sons.

Hu, F. and Zhang, L.-X. (2004). Asymptotic properties of doubly adaptive biased coin designs for multi-treatment clinical trials. *Annals of Statistics* **32,** 268–301.

Hu, F., Zhang, L.-X., and He, X. (2009). Efficient randomized-adaptive designs. *Annals of Statistics* **37,** 2543–2560.

Ivanova, A. (2003). A play-the-winner type urn design with reduced variability. *Metrika* **58,** 1–13.

Johnson, N. L. and Kotz, S. (1977). *Urn Models and Their Application.* Wiley, New York.

Korn, E. L. and Freidlin, B. (2011). Outcome–adaptive randomization: is it useful? *Journal of Clinical Oncology* **29,** 771–776.

Lee, J. J., Chen, N., and Yin, G. (2012). Worth adapting? Revisiting the usefulness of outcome-adaptive randomization. *Clinical Cancer Research* **18,** 4498–4507.

Melfi, V., Page, C., and Geraldes, M. (2001). An adaptive randomized design with application to estimation. *Canadian Journal of Statistics* **29,** 107–116.

Rosenberger, W. F. and Hu, F. (2004). Maximizing power and minimizing treatment failures in clinical trials. *Clinical Trials* **1,** 141–147.

Rosenberger, W. F. and Lachin, J. M. (2016). *Randomization in Clinical Trials: Theory and Practice.* Wiley, 2nd edition.

Rosenberger, W. F., Stallard, N., Ivanova, A., Harper, C. N., and Ricks, M. L. (2001). Optimal adaptive designs for binary response trials. *Biometrics* **57,** 909–913.

Scott, S. L. (2010). A modern Bayesian look at the multi-armed bandit. *Applied Stochastic Models in Business and Industry* **26,** 639–658.

Smith, A. and Villar, S. S. (2018). Bayesian adaptive bandit-based designs using the gittins index for multi-armed trials with normally distributed endpoints. *Journal of Applied Statistics* **45,** 1052–1076.

Sverdlov, O. and Rosenberger, W. F. (2013). On recent advances in optimal allocation designs in clinical trials. *Journal of Statistical Theory and Practice* **7,** 753–773.

Thall, P., Fox, P., and Wathen, J. (2015). Statistical controversies in clinical research: scientific and ethical problems with adaptive randomization in comparative clinical trials. *Annals of Oncology* **26,** 1621–1628.

Thall, P. F. and Wathen, J. K. (2007). Practical Bayesian adaptive randomisation in clinical trials. *European Journal of Cancer* **43,** 859–866.

Trippa, L., Lee, E. Q., Wen, P. Y., Batchelor, T. T., Cloughesy, T., Parmigiani, G., et al. (2012). Bayesian adaptive randomized trial design for patients with recurrent glioblastoma. *Journal of Clinical Oncology* **30,** 3258–3263.

Tymofyeyev, Y., Rosenberger, W. F., and Hu, F. (2007). Implementing optimal allocation in sequential binary response experiments. *Journal of the American Statistical Association* **102,** 224–234.

Villar, S. S., Bowden, J., and Wason, J. (2015). Multi-armed bandit models for the optimal design of clinical trials: benefits and challenges. *Statistical Science* **30,** 199.

Villar, S. S., Wason, J., and Bowden, J. (2015a). Response-adaptive randomization for multi-arm clinical trials using the forward looking Gittins index rule. *Biometrics* **71,** 969–978.

Wason, J. M. S. and Trippa, L. (2014). A comparison of Bayesian adaptive randomization and multi-stage designs for multi-arm clinical trials. *Statistics in Medicine* **33,** 2206–2221.

Wathen, J. K. and Thall, P. F. (2017). A simulation study of outcome adaptive randomization in multi-arm clinical trials. *Clinical Trials* **14,** 432–440.

Wei, L. (1979). The generalized polya's urn design for sequential medical trials. *The Annals of Statistics* **7,** 291–296.

Wei, L. and Durham, S. (1978). The randomized play-the-winner rule in medical trials. *Journal of the American Statistical Association* **73,** 840–843.

Williamson, S. F., Jacko, P., Villar, S. S., and Jaki, T. (2017). A Bayesian adaptive design for clinical trials in rare diseases. *Computational Statistics & Data Analysis* **113,** 136–153.

Yin, G., Chen, N., and Jack Lee, J. (2012). Phase II trial design with Bayesian adaptive randomization and predictive probability. *Journal of the Royal Statistical Society: Series C* **61,** 219–235.

Zhang, L. and Rosenberger, W. F. (2006). Response-adaptive randomization for clinical trials with continuous outcomes. *Biometrics* **62,** 562–569.

Zhang, L. X., Chan, W. S., Cheung, S. H., and Hu, F. (2007). A generalized urn model for clinical trials with delayed responses. *Statistica Sinica* **17,** 387–409.
